# Supplementary material for: Recoverability of causal effects under presence of missing data: a longitudinal case study
Source: Biostatistics. 2024 Nov 16;26(1):kxae044. doi: 10.1093/biostatistics/kxae044 (PMC7617375; doi:10.1093/biostatistics/kxae044)
Supplement: kxae044_Supplementary_Data [file kxae044_supplementary_data.zip › 1650117mhjtpfkghzcddgmsdgnjbrrdmftrfsbq/oup-authoring-template.pdf]

Biostatistics Vol. , No. , pp. 1–23
doi: DOI HERE
Advance Access Publication Date: Day Month Year
Paper

Recoverability of Causal Effects in a Longitudinal Study under Presence of Missing Data

Anastasiia Holovchak,[1,*] Helen McIlleron,[2] Paolo Denti[2] and Michael Schomaker[3,4]

[1]Seminar for Statistics, ETH Zurich, Switzerland, [2]Division of Clinical Pharmacology, Department of Medicine, University of Cape Town, South Africa, [3]Department of Statistics, Ludwig-Maximilians University Munich, Germany and [4]Centre for Infectious Disease Epidemiology and Research, University of Cape Town, South Africa

[*]Anastasiia Holovchak anastasiia.holovchak@stat.math.ethz.ch
FOR PUBLISHER ONLY Submitted Date Month Year; Revised Date Month Year; revised version accepted Date Month Year

ABSTRACT

Missing data in multiple variables is a common issue. We investigate the applicability of the framework of graphical models for handling missing data to a complex longitudinal pharmacological study of children with HIV treated with an efavirenz-based regimen as part of the CHAPAS-3 trial. Specifically, we examine whether the causal effects of interest, defined through static interventions on multiple continuous variables, can be recovered (estimated consistently) from the available data only. So far, no general algorithms are available to decide on recoverability, and decisions have to be made on a case-by-case basis. We emphasize sensitivity of recoverability to even the smallest changes in the graph structure, and present recoverability results for three plausible missingness directed acyclic graphs (m-DAGs) in the CHAPAS-3 study, informed by clinical knowledge. Furthermore, we propose the concept of "closed missingness mechanisms" and show that under these mechanisms an available case analysis is admissible for consistent estimation for any type of statistical and causal query, even if the underlying missingness mechanism is of missing not at random (MNAR) type. Both simulations and theoretical considerations demonstrate how, in the assumed MNAR setting of our study, a complete or available case analysis can be superior to multiple imputation, and estimation results vary depending on the assumed missingness DAG. Our analyses are possibly the first to show the applicability of missingness DAGs (m-DAGs) to complex longitudinal real-world data, while highlighting the sensitivity with respect to the assumed causal model.

KEYWORDS: missing data, missingness DAG, causal effect, longitudinal data

INTRODUCTION

Missing data are a common issue in biomedical research. In particular, longitudinal epidemiological studies where data are collected consecutively at several points in time, tend to suffer from missing data on multiple variables. One common assumption which is often made regarding the missing data mechanism is the so-called *missing at random* (MAR) assumption. If this assumption holds, statistical methods for consistent estimation are available, for example multiple imputation or inverse probability weighting (Rubin, 1976; Mohan and Pearl, 2014).

However, if multiple variables are missing simultaneously, it is often difficult to justify whether the MAR assumption holds. This is both because the classic MAR definition is event- rather than variable-based, and arguing for or against conditional independencies of *events* is practically challenging, especially in longitudinal settings (Mohan and Pearl, 2021). Moreover, even though MAR is the weakest known condition under which the missingness process can be ignored (i.e., dealt with using the observed data), it is only a sufficient, but

© The Author 2022. Published by Oxford University Press. All rights reserved. For permissions, please e-mail: journals.permissions@oup.com

not a necessary condition for unbiased estimation; this means that under a *missing not at random* (MNAR) scenario, it is unclear if and how a target estimand can be recovered (estimated consistently).

To tackle these and other challenges, Mohan et al. (2013) and Mohan and Pearl (2021) proposed an alternative causal graph-based framework, in which knowledge and assumptions about *reasons* for missingness are encoded in relationships between *variables* and missing data indicators. This framework is very general and can be used to evaluate whether estimands can be recovered given a correct causal missingness model (missingness directed acyclic graph, m-DAG).

However, currently there is no general algorithm which can be used to establish recoverability for arbitrary settings. Therefore, identification and estimation strategies have to be developed on a case-by-case basis for each particular scenario. This poses the question of how useful causal m-DAGs are practically.

Moreno-Betancur et al. (2018) have convincingly argued that one should thus develop canonical m-DAGs for recurring settings, such as for point-exposure study designs in epidemiology where missing data in outcome, exposure, and confounders are caused by some "standard" mechanisms. They demonstrated the usefulness of this approach in a study investigating the relationship between maternal mental illness and child behavior.

While causal missingness graphs, and their canonical versions, are a major advancement for causal inference research under missing data, its actual applicability has yet to be demonstrated in complex longitudinal studies. It is unclear how well knowledge on missing data can be actually collected, then integrated in a realistic causal graph, and how difficult the mathematical exercise of establishing identification and recoverability results in such a complex, yet realistic setting is. Can m-DAGs make their way from blackboards to actual applications?

Additionally, we conduct simulation studies aligned with the data example to, firstly, verify the theoretical results on the recoverability of the desired causal query, and secondly, to quantify the extent of bias in settings of special interest. Our simulations and theoretical considerations demonstrate that causal diagrams help to explicitly guide the process of decision-making about whether a parameter of interest can be consistently estimated from the available data. In practice, MAR is often assumed and multiple imputation is performed, which in many cases may lead to biased estimation results. On the other hand, we demonstrate that even in complex longitudinal study settings, there are MNAR scenarios for which available case analysis leads to valid estimation results, whereas multiple imputation does not.

We sought to answer these questions by applying the framework proposed by Mohan and Pearl (2021) to answer a topical question in HIV-related pharmacoepidemiology, which is explained below in Section 2. We i) investigate how clinical knowledge on reasons for our missing data can be best collected and integrated into a realistic causal missingness graph, ii) derive recoverability results for our target estimand, iii) discuss estimation strategies for it, iv) evaluate the sensitivity of our results with respect to the assumed causal model and v) seek for structures that may be helpful in establishing results for future studies.

This paper is structured as follows. We introduce the motivating question in Section 2, followed by the theoretical framework in Section 3. In Section 4, analyse the data from the complex longitudinal pharmacological study CHAPAS-3, followed by the simulation study in Section 5. We conclude in Section 6.

MOTIVATING STUDY

Our motivating data analysis comes from the Children with HIV in Africa–Pharmacokinetics and Adherence/Acceptability of Simple antiretroviral regimens randomized trial (CHAPAS-3). The study enrolled 478 children with HIV, between 1 month and 13 years of age, in 4 sites in Uganda and Zambia (Mulenga et al., 2016). Children enrolled into the trial received combined antiretroviral therapy, i.e. one non-nucleoside reverse transcriptase inhibitor (efavirenz or nevirapine) and two nucleoside reverse transcriptase inhibitors (abacavir, stavudine or zidovudine –which were the randomized components– and lamivudine).

We are interested in determining target concentrations using data from 125 children treated with efavirenz (EFV). Efavirenz is used not only in children, but also adults, though dosing recommendations (between 200 and 600mg) depend on weight and age. Due to their different metabolic profiles and adherence patterns, patients with the same efavirenz dose may have different concentrations, conferring different protection against viral replication. Knowledge about concentrations is often used to derive dosing recommendations using population PK models (Bienczak et al., 2016). It is typically suggested that EFV concentrations between 1 and 4 mg/L should be achieved (at 12h after the dose was given, C12h). This is because lower concentrations may be insufficient to guarantee viral suppression and thus effective treatment, while higher concentrations may lead to toxicity negatively affecting the central nervous system. Our target estimand is thus the causal concentration-response curve (CCRC) at each follow-up visit, i.e. *we are interested in how the counterfactual probability of viral failure at time t varies as a function of possible prior concentration trajectories.*

Our analysis makes use of the data from Bienczak et al. (2016). We recently discussed statistical approaches on estimating the CCRC Schomaker et al. (2023), but did not discuss aspects of missing data. In this manuscript, we extend the above study by developing a causal missingness graph (informed by the pediatrician's

and trial team's knowledge) and derive identification and estimation approaches for our estimand of interest. More details on the analysis are given in Section 4.

FRAMEWORK

Missingness DAGs provide a natural extension of the causal DAGs under the presence of missing data. Consider a DAG $G = (V, E)$ with a set of nodes V, $|V| = n$, which can be separated into two subsets V_o and V_m, corresponding to sets of *completely observed* and *partially observed* variables. For each variable X_i, $i \in \{1, ..., n\}$, from the subset V_m, a binary missingness indicator variable M_{X_i}

$$M_{X_i} = \begin{cases} 1 & \text{if } X_i \text{ is missing,} \\ 0 & \text{otherwise} \end{cases}$$

is introduced in the missingness DAG to depict causal relationships with other relevant variables. In the following, we refer to the missingness indicator variable's set as M. Additionally, the setup allows for existence of a latent (unmeasured) variable set U.

In this work, we make use of the framework introduced by Mohan and Pearl (2021). We refer to G_c as the *complete-DAG (c-DAG)* (as in Moreno-Betancur et al. (2018)), and G as the *missingness DAG (m-DAG)*. The c-DAG G_c includes only the variable set $V := V_o \cup V_m \cup U$ that is relevant for identification considerations with respect to the (causal) parameter of interest (assuming no missingness in the variables in V_m). The m-DAG G, however, also includes the set of missingness indicators M, and may additionally consist of a set of auxiliary variables, denoted as Z, with variables in Z causing missingness in the variables in V_m, but not being a cause of the variables from the set V. Note that variables from both V and Z may be a cause of missingness in the variables from V_m.

Mohan et al. (2013), Mohan and Pearl (2021) show that causal diagrams can be used as a powerful tool for identification and classification of missingness mechanisms. It is an important finding that the conventional taxonomy of missing data (Rubin, 1976) can be translated (with some minor changes) into the context of causal diagrams, which the focus of this work lies on. However, it is essential to mention that the missingness taxonomy definitions proposed by Rubin (1976) and Mohan et al. (2013) are, in general, not equivalent. Note that Rubin's framework of missing data is defined on the *record-based level*, which makes it particularly difficult to apply in practice. Another issue that is often ignored is that the 'realized' MAR definition as proposed by Rubin (1976) is not required to hold for all possible values of the missingness indicator M, but only for those present in the data. This makes clear that such a kind of definition does not hold for a data-generating distribution in general, but only for a single data sample, as different missing data patterns may arise for samples resulting from re-running of the experiment (Schafer and Graham, 2002; Tian, 2015). To overcome this issue, Seaman et al. (2013) distinguishes between two types of MAR - *realized* MAR and *everywhere* MAR. The last one is a 'stronger' definition in the sense that conditional missingness distribution relates to the missing entries for all realized and unrealized patterns of missing data, and not only to the one specific data sample which has been observed.

Mohan et al. (2013) propose an alternative framework of missing data taxonomy, which is based on graphical models. The two main advantages of the graph-based method are an explicit encoding of dependencies on the variable level (and not record-based), and also depiction of causal mechanisms which are causing missingness (Tian, 2015). Note that m-DAGs represent both the data generating mechanism and the missing data mechanism, both in a causal manner.

Let G be an m-DAG over a set of variables $V_o \cup V_m \cup U \cup M$, where V_o and V_m denote the sets of completely and partially observed variables, correspondingly, U is a set of latent (unobserved) variables, and M is the set of missingness indicators. The missingness mechanism is commonly characterized in terms of the conditional distribution of M given the data (V_o, V_m, U), say $p(M|V_o, V_m, U)$. It has to be assumed that any missingness indicator from the set M is not a parent of any variable from $V_o \cup V_m \cup U$. We emphasize once more that in the graph-based missing data framework, we work with variable-based and not the record-based definitions of the missingness mechanisms.

1. **Missing completely at random (MCAR).** If missingness occurs randomly and is assumed not to be caused by any variable in the model, missing or observed, we write

$$p(M|V_o, V_m, U) = p(M),$$

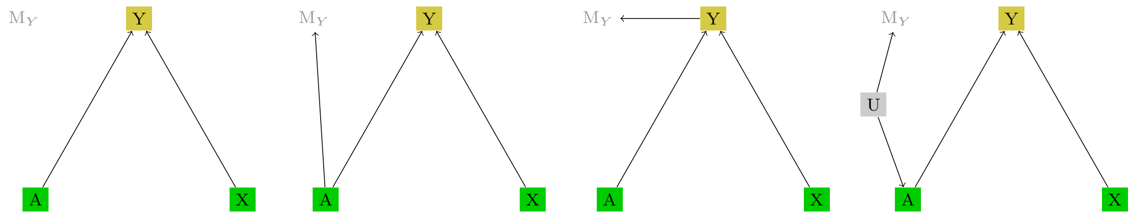

(a) MCAR (b) MAR (c) MNAR^a (d) MNAR^b

Fig. 1. Example m-DAGs depicting three different types of missingness mechanisms. Fully and partially observed variables have been depicted in green and yellow squares, respectively. We assume Y to be the disease indicator, A the type of treatment and X the patient age. M_Y is the missingness indicator for outcome Y.

which means that the conditional distribution of the missingness mechanisms given the variables in the data set and, possibly, also the latent variables, is equal to the marginal distribution of the missingness mechanism. This corresponds to the (unconditional) independence statement $(V_o, V_m, U) \perp\!\!\!\perp M$.
In terms of an m-DAG, if the joint distribution $p(V_o, V_m, U, M)$ is faithful with respect to the graph G, this means that there are *no edges between the M variables and variables in V_o and V_m*, and parents of missingness indicator variables from M can only be other variables from M.
A commonly used example of the MCAR mechanism is an accidental technical error in an electronic medical record, which leads to a loss of data on the disease indicator, compare Figure 1a.

2. **Missing at random (MAR).** In this case, the missingness mechanism is assumed to depend on the fully observed variables only, but is not allowed to depend on the missing data values or latent variables. In terms of the conditional distribution of M, this corresponds to

$$p(M|V_o, V_m, U) = p(M|V_o).$$

The conditional independence statement that holds in this case is $(V_m, U) \perp\!\!\!\perp M|V_o$, which in terms of m-DAGs (if the joint distribution is faithful to G) means that *variables from M are not allowed to have any parents from the sets V_m or U, but only from V_o and other M variables.*
For example, if a disease indicator is missing for some patients, and missingness for some reason depends on the completely observed treatment only, one says that the missing data underlies the MAR mechanism, as depicted in the example in Figure 1b.

3. **Missing not at random (MNAR).** This category of missingness is most general. Data that cannot be classified as MCAR or MAR fall in the MNAR category. In this case, the conditional distribution of M, $p(M|V_o, V_m, U)$, cannot be simplified. In an m-DAG (again, assuming faithfulness), if at least one M variable has a parent which is a latent variable U or is any of the partially observed variables from the set V_m, then the missing data mechanism is MNAR.
A common example of MNAR is when a variable is causing its own missingness, e.g., if the missingness of the disease status entry depends on the disease status itself, as this is the case for the m-DAG in Figure 1c. For instance, the presence of a disease is likely to increase the chances of disease status being recorded due to the associated medical follow-ups and tests.
Another practically very relevant example is presented in Figure 1d. It is showing a case where missingness is driven by latent variables, which may potentially also be a cause for an observed variable. For example the socio-economic status (SES) may not be recorded in a medical study, but has an impact on both, the missingness in the disease indicator due to reduced healthcare visits, and the treatmentthe type of treatment an individual receives, since those with higher SES may have access to better healthcare facilities and more advanced treatment options.

Note that both missing data taxonomies are equivalent if i) observations are independent and ii) missingness indicators are conditionally independent (Schomaker, 2020). In this case, the conditional distribution from the graph-based MAR definition can be written as a product of conditional distributions for single missingness indicators as well as for single observations, and this then leads to the equivalence of both definitions.

Recoverability of Target Quantities

In general, recoverability is a property of the target quantity/parameter θ (and the probability distribution P_X) which states whether this quantity can be estimated consistently from the available data. To decide on

recoverability, one needs to know the independence statements which hold in the joint distribution. However, under the Markovness and faithfulness assumptions, all conditional independence statements present in the data can be directly read off from the m-DAG G, and recoverability can be therefore considered as a property of the pair $\{\theta, G\}$ (Mohan and Pearl, 2021). Note that the term *identifiability* is used when assessing whether a causal query can be expressed as a function of the observational distribution, whereas the *recoverability* concept is used to describe whether a parameter (not necessarily in a causal context) can be expressed as a function of the available data distribution (Moreno-Betancur et al., 2018).

Moreno-Betancur et al. (2018) describe three main conditions, required for recoverability of a target parameter θ - consistency and well-defined interventions, positivity, and conditional independence conditions. In this context, consistency is a property of the central relevance. It says that the factual treatment value coincides with the counterfactual outcome.

It has to be emphasized that many types of target parameters θ may be of interest, and different missingness patterns may occur, which makes it impossible to derive a general automatized algorithm allowing decision about recoverability of any specific θ. Therefore, the authors work on 'canonical' scenarios which are most typical for epidemiological studies. Mohan et al. (2013), Mohan and Pearl (2021) present some theoretical results on recoverability of joint and conditional distributions. In this work, we only briefly provide intuition on how recoverability works in general, focusing on aspects that are relevant for our own identification strategies. The main goal is to exploit conditional independencies between the variables of interest and the missingness indicators (which can be read off from the d-separation statements which hold in the graphs) in order to be able to condition on the missingness indicator variables (i.e., on $M_i = 0$ for some i). This way, observed variable values will be sufficient for consistent estimation of the target quantity.

In the following, we focus on the recoverability of causal effects. Indeed, necessary and sufficient conditions exist for recoverability of causal effects (Mohan and Pearl, 2021). Under the presence of missing data, a *necessary condition for recoverability* of a causal effect is the identifiability of this effect from the c-DAG of interest. The causal effect is *identifiable* from a graph G if the interventional distribution can be determined uniquely from the observed-data distribution alone (Pearl, 2009; Tian and Pearl, 2002).Shpitser and Pearl (2006) provide a sound and complete algorithm for conditional causal effect identification (IDC), which, for any causal effect, can be used for determining the identifiability, but also for generation of an expression for the interventional distribution in the case of an identifiable effect. Tikka and Karvanen (2017) implemented the IDC algorithm in the R-package `causaleffect`. Using the IDC algorithm, we get an estimand of the causal query whenever identifiability holds for the causal effect. In turn, a sufficient condition for recoverability is that the (identified) estimand is recoverable from the missingness graph. This, as mentioned before, has to be decided on a case-by-case basis.

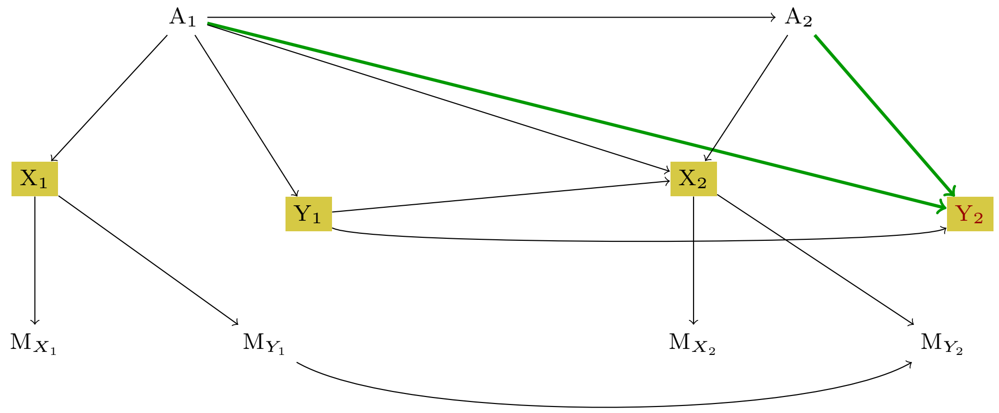

Fig. 2. m-DAG depicting MNAR missingness mechanism in a simple longitudinal study. We denote health outcomes at two study time points as Y_1 and Y_2, A_1 and A_2 correspond to sequential treatment variables, X_1 and X_2 are the side effects.

Next, an example of recoverability procedure of a causal effect for a simple longitudinal study with two measurement points is presented. The example is inspired by Mohan and Pearl (2021, Section 3.5). Consider

the m-DAG in Figure 2, which is a model of a simple two time point longitudinal study with attrition. The variables A_1 and A_2 correspond to sequential treatment, X_1 and X_2 are the side effects, and Y_1 and Y_2 model some health outcomes. The causal effect of interest is $P(Y_2; do(A_1, A_2))$, which is the effect of the two sequential treatments on the outcome at the second study time point. We can see that the partially observed variables X_1 and X_2 are causing their own missingness, which means that the missingness mechanism is of the MNAR type. However, even in this case, it can be shown that the causal effect of interest is recoverable using sequential factorization (Mohan and Pearl, 2021). First, with help of the IDC algorithm an expression for identifying the causal effect from the observable data is provided (for the case as if there has been no missingness, which is indicated in terms of potential outcomes):

$$P(Y_2^{M=0}; do(A_1, A_2)) = \sum_{Y_1} P(Y_2^{M=0}|A_1, A_2, Y_1^{M=0}) \times P(Y_1^{M=0}|A_1).$$

Note that the potential outcome notation $(\cdot)^{M=0}$ is explicitly used only for the variables observed incompletely. In the next step, it has to be decided on the recoverability of the two conditional distributions $P(Y_2^{M=0}|A_1, A_2, Y_1^{M=0})$ and $P(Y_1^{M=0}|A_1)$.

$$\begin{aligned} P(Y_2^{M=0}|A_1, A_2, Y_1^{M=0}) &= P(Y_2^{M=0}|A_1, A_2, Y_1^{M=0}, M_{Y_1} = 0, M_{Y_2} = 0) && (Y_2 \perp\!\!\!\perp \{M_{Y_1}, M_{Y_2}\}|A_1, A_2, Y_1) \\ &= P(Y_2|A_1, A_2, Y_1, M_{Y_1} = 0, M_{Y_2} = 0) && \text{(by consistency)} \end{aligned}$$

$$\begin{aligned} P(Y_1^{M=0}|A_1) &= P(Y_1^{M=0}|A_1, M_{Y_1} = 0) && (Y_1 \perp\!\!\!\perp M_{Y_1}|A_1) \\ &= P(Y_1|A_1, M_{Y_1} = 0) && \text{(by consistency)} \end{aligned}$$

Formally, it is true that $\{A_1, A_2, Y_1\}$ is the minimal set for which

$$Y_2 \perp\!\!\!\perp \{X_1, X_2\}|A_1, A_2, Y_1.$$

Analogously, A_1 is the minimal set for which holds

$$Y_1 \perp\!\!\!\perp X_1|A_1.$$

Further, $P(Y_2|A_1, A_2, Y_1)$ satisfies $Y_2 \perp\!\!\!\perp \{M_{Y_1}, M_{Y_2}\}|A_1, A_2, Y_1$, whereas for $P(Y_1|A_1)$ it holds true $Y_1 \perp\!\!\!\perp M_{Y_1}|A_1$. Then, applying sequential factorization technique, both factors can be recovered as $P(Y_2|A_1, A_2, Y_1, M_{Y_1} = 0, M_{Y_2} = 0)$ and $P(Y_1|A_1, M_{Y_1} = 0)$, correspondingly.

This shows that the causal effect of interest can be recovered from the available data only despite the fact that the underlying missingness mechanism is MNAR.

After a causal effect of interest has been identified as a function of the observed data, (and after it has been further ensured that and how it can be recovered under the presence of missing data), the causal effect can be estimated using the common techniques, e.g., (parametric) G-computation (Robins, 1986), inverse probability weighting (Hernán and Robins, 2006; Hernan and Robins, 2020) or targeted maximum likelihood estimation (TMLE) (van der Laan and Rose, 2011).

Closed Missingness Mechanism

Next, an interesting special case of missingness mechanism, which we refer to as *closed missingness mechanism*, is discussed. We present the recoverability results for joint and conditional distributions under the closed missingness mechanism.

Definition 1 (Closed missingness) Consider a c-DAG G_c with the node set $V = \{X_1, X_2, ..., X_n\}$, $|V| = n$. Without loss of generality, assume that $V_o = \{X_1, ..., X_k\}$ and $V_m = \{X_{k+1}, ..., X_n\}$ for some $k \leq n$. The corresponding m-DAG consists of the set V, the missingness indicator variable set $M = \{M_{X_{k+1}}, ..., M_{X_n}\}$, and possibly a variable set Z containing auxiliary variables that are causes of missingness. The missingness mechanism is called closed if there is no path between any $V_i \in V$ and any $M_{V_i} \in M$, $i \in \{k+1, ..., n\}$.

In other words, the missingness mechanism is closed if only the auxiliary variables from Z are causes of missingness. From a practical standpoint, this type of missingness mechanism is common in clinical, epidemiological, and pharmacological studies. The variables of interest in these studies are often of biological and

medical nature (e.g., medication/therapy type, medication dose, blood values), whereas the causes of missingness in such variables are typically related to external factors such as technical issues with medical devices and the socioeconomic status of study participants.

Corollary 1 *Consider an m-DAG G depicting a closed missing mechanism, and the general setting as in Definition 1. The joint distribution* $P(V_o, V_m)$*, and therefore also any marginal or conditional distribution, is recoverable from the available cases.*

Proof As there is no path between any $V_i \in V, i \in \{1, ..., n\}$, and any $M_{V_i} \in M$, $i \in \{k+1, ..., n\}$, V_i and M_{V_i} are d-separated for any i without conditioning on any other variables, and therefore $V_i \perp\!\!\!\perp M_{V_i}$ holds true. □

Note that the variables from Z may also be completely and incompletely observed, or even unobserved or latent, but this plays no role for the variables of interest from the set V.

The concept of a 'closed missingness mechanism' can lead to useful practical rule of thumb, especially under MNAR. If a researcher determines that missingness is likely caused by unmeasured factors, they may initially conclude that the data are MNAR, making both imputation and available case analyses invalid. However, by sketching a c-DAG and m-DAG (incorporating missingness indicators), if no arrows are found from the c-DAG to the m-DAG, they can conclude that a available or complete case analysis is valid, despite MNAR. Intuitively, if the causes of missing values are unrelated to the variables necessary for identification, then relying solely on complete cases might be preferable to imputation.

This approach underscores a crucial practical insight: even in the presence of MNAR, if the missingness mechanism is closed, it does not impact the validity of complete case analysis. Thus, researchers can confidently use complete case data when the missingness indicators are isolated from the primary variables of interest.

DATA ANALYSIS

Study setting, measurements and estimand

As described in Section 2, our data comes from the study of Bienczak et al. (2016) and includes the 125 children with HIV who were enrolled in the CHAPAS-3 trial and were treated with an efavirenz-based regimen. We consider the trial's follow-up visits at 6, 36, 48, 60, and 84 weeks.

Our scientific goal is to estimate causal concentration response-curves. That is, we are interested in the counterfactual probability of viral load (VL) > 100 copies/ml (which is considered to be a viral failure) at 36 and 84 weeks if children had efavirenz concentrations (12h after dose) of x mg/L at each follow-up visit, where x ranges from 0 to 10 mg/L. Missing data in the outcome (VL), efavirenz exposure (EFV) and time-dependent confounders (weight, adherence) complicates this exercise.

Measured baseline variables include sex, age, the nucleoside reverse transcriptase inhibitors drug (NRTI) component of the treatment regimen and weight. Moreover, we include knowledge on the metabolism status ("genotype", slow, intermediate, extensive) related to the single nucleotide polymorphisms in the CYP2B6 gene, which is relevant for metabolizing evafirenz and directly affects its concentration in the body. Measured follow-up variables are weight, adherence (measured through memory caps, MEMS) and dose.

Clinical assessments were made at every visit, viral loads at all time points except week 6, efavirenz levels at all assessments other than week 48, and assessment of adherence through MEMS mostly at weeks 36, 48 and 60 as both funding constraints and practical considerations did not allow its consecutive implementation.

Development of the causal model

Figure 3 contains our proposed causal model. The causal graph contains 1) variables which are important to identify the effect of interest (i.e. the effect of EFV on viral failure, c-DAG, bottom). We summarized 2) clinician's knowledge on why data are possibly missing in a m-DAG using binary missingness indicator variables (top, pink shading) for relevant variables with missing data (EFV, elevated viral load (VL), adherence (MEMS), weight).

c-DAG

The c-DAG summarizes our knowledge and assumptions, described in more detail Schomaker et al. (2023). Briefly, the main cause of viral failure is subtherapeutic efavirenz concentration ($\text{EFV}_t \rightarrow \text{VL}_t$). The concentration itself depends on the administered dose ($\text{Dose}_t \rightarrow \text{EFV}_t$), adherence to treatment ($\text{MEMS}_t \rightarrow \text{EFV}_t$) and how fast the drug is cleared, determined - amongst other - by the 516G and 983T polymorphisms in the CYP2B6 gene (Genotype $\rightarrow \text{EFV}_t$). Both weight and MEMS are assumed to be time-dependent confounders. Co-morbidities (CoMo), which we defined to include tuberculosis, pneumonia and other AIDS-defining events

are reflected in the DAG, although they are expected to be less frequent in our population, as children with active infections, being treated for tuberculosis and with severe laboratory abnormalities at screening, were not enrolled into the study, and most children were in relatively good health (e.g. median CD4 cell percentage is 19%).

m-DAG

Main m-DAG (G_{main}): The development of the missingenss causal model is a novel contribution of our paper. Reasons for missing data were obtained from the trial team and the paediatricians. Those reasons are represented by arrows leading into the missingness indicators and are i) technical issues with the memory caps (e.g., breaking of containers) or in obtaining or analysing a blood sample (TI, unmeasured); ii) missed visits (MV), which are likely related to socio-economic status of the caregiver (SES, measured), beliefs and attitudes towards medicine (BMQ, measured) and other behavioural factors (BHV, unmeasured). As almost all children depend on their caregiver to arrive at their appointment, we assume that the children's age does not affect the probability of a missed visit.

Sensitivity of the main causal missingness model (G_{alt1}): While the clinicians did not report any other possible reasons for missingness, we added another speculative reason for missed visits to explore the implications of deviations from the assumed causal model: health status of the child, captured indirectly through elevated viral load. This possible reason is represented by the blue dashed arrows in the m-DAG (G_{alt1}). In the discussion, we also briefly discuss another m-DAG (G_{alt2}), where G_{main} is extended with additional arrows SES $\rightarrow$ MEMS$_t$, $t \in \{6, 36, 48, 60, 84\}$.

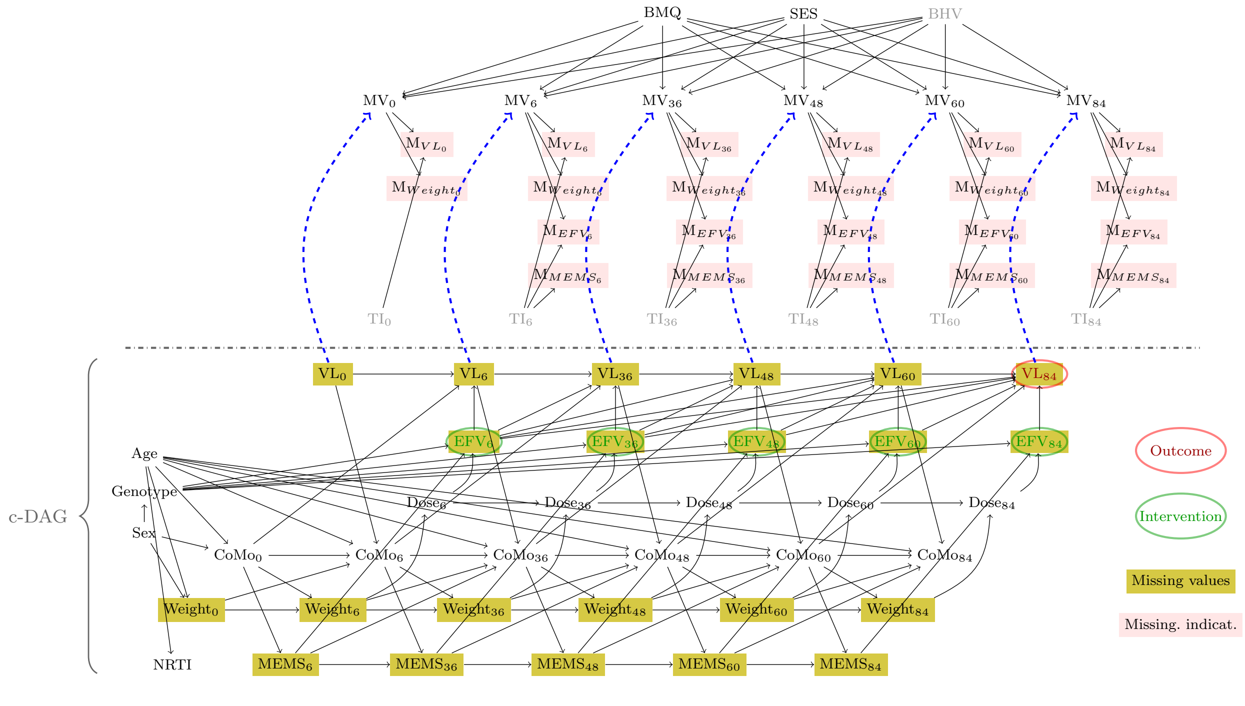

Fig. 3. m-DAG. Blue dashed arrows distinguish between G_{main} (without blue arrows) and G_{alt1} (with blue arrows). The subgraph below the gray dashed line represents the corresponding c-DAG. Unmeasured variables are presented in gray.

Following the introduced notations, and under the assumption of G_{main} or G_{alt1}, the sets of completely and partially observed variables, auxiliary variables and missingness indicators are defined as following:

- $V_o = \{Age, Genotype, Sex, NRTI, CoMo_t, Dose_t\}$
- $V_m = \{MEMS_t, Weight_t, EFV_t, VL_t\}$
- $M = \{M_{VL_t}, M_{Weight_t}, M_{EFV_t}, M_{MEMS_t}\}$

- $Z = \{TI_t, MV_t, BMQ, SES, BHV\}$.

Note that under G_{alt2} (G_{main} with additional arrows SES → MEMS$_t$), BHV becomes a part of the c-DAG.

Identifiability and Recoverability of Causal Effects

To make a decision on recoverability of a causal query of interest, we first need to find out whether the query is identifiable. Provided the identifiability holds true, the main idea of recoverability is to transform the partially observed variables from the identified expression into observables with help of d-separation statements resulting from the m-DAG.

In this work, we aim to estimate the causal effect of the efavirenz drug concentration in plasma of children with HIV, measured and controlled over a particular time period, on viral failure.

Particularly, we focus on two causal effects,

$$\theta_{36} := P(VL_{36} = 1;\ do(EFV_6 := a, EFV_{36} := a)) \tag{1}$$

and

$$\theta_{84} := P(VL_{84} = 1; do(EFV_6 := a, EFV_{36} := a, EFV_{48} := a, EFV_{60} := a, EFV_{84} := a)), \tag{2}$$

corresponding to the probability of viral failure after 36 and 84 weeks under a fixed intervention on the plasma concentration of efavirenz drug at each previous up to current study time point. Note that for assessment of recoverability of the causal effects, only variables from the previous up to current study time point are considered, and we therefore focus on the corresponding subgraph of the 'full' m-DAG in Figure 3 containing the variables up to and including the 36-th study week when deciding about identifiability and recoverability of θ_{36}.

We first consider the m-DAG G_{main} from Figure 3, ignoring the dashed blue lines from VL_t to MV_t, $t \in \{0, 6, 36, 48, 60, 84\}$. Note that according to the taxonomy of the missingness mechanisms proposed byMohan et al. (2013), the missingness is of MNAR type because TI_t, $t \in \{0, 6, 36, 48, 60, 84\}$, are unobserved variables directly causing missingness. Under Rubin's definition the data would also be MNAR because units may exhibit missing values due to unmeasured behavioral factors (BHV).

In the first step, we have to work on identifiability of the causal effect of interest. We carry this out through two approaches. Our first proposal is the application of the IDC algorithm. Applying rules of *do-calculus* therefore leads to the causal effect identifiable in terms of the observed data distribution as if there has been no missingness in any variable relevant for identification:

$$\begin{aligned}\theta_{36} = \sum_{\substack{Age, Sex, CoMo_0, Weight_0,\\ VL_0, CoMo_6, VL_6}} & P(VL_{36} = 1 | Age, Sex, CoMo_0, Weight_0, Genotype, MEMS_6, Weight_6, Dose_6,\\ & EFV_6 = a, VL_0, CoMo_6, VL_6, MEMS_{36}, Weight_{36}, Dose_{36}, EFV_{36} = a)\\ & P(VL_6 | Age, Sex, CoMo_0, Weight_0, Genotype, MEMS_6, Weight_6,\\ & Dose_6, EFV_6 = a, VL_0)\\ & P(CoMo_6 | Age, Sex, CoMo_0, Weight_0, Genotype, VL_0)\\ & P(Weight_0 | Age, Sex) P(CoMo_0 | Age, Sex)\\ & P(Sex) P(Age) P(VL_0).\end{aligned} \tag{3}$$

The corresponding identifiability result for θ_{84} based on IDC algorithm is in Appendix A.1.

There may be other identifiability results, based on different factorizations. For example, in our second proposal, we apply Pearl's (generalized) back door-criterion to determine a sufficient adjustment set and then simply apply Robins' g-formula factorization to it. Such a factorization may involve more conditional distributions that have to be estimated compared to the first approach. For the above estimand, θ_{36}, a valid

factorization would be (see, e.g., Hernán and Robins (2020, Chapters 19 and 21)):

$$
\begin{aligned}
\theta_{36} = \sum_{\substack{Age, Genotype, Sex, NRTI, Weight_0, VL_0 \\ Weight_6, MEMS_6, Weight_{36}, MEMS_{36}}} & P(VL_{36} = 1 | EFV_6 = a, EFV_{36} = a, Age, Genotype, Sex, NRTI, \\
& Weight_0, Weight_6, MEMS_6, Weight_{36}, MEMS_{36}, VL_0, VL_6) \\
& P(Weight_{36} | EFV_6 = a, Age, Genotype, Sex, NRTI, \\
& VL_0, VL_6, Weight_0, Weight_6, MEMS_6) \\
& P(MEMS_{36} | EFV_6 = a, Age, Genotype, Sex, NRTI, \\
& VL_0, VL_6, Weight_0, Weight_6, MEMS_6) \\
& P(VL_6 | EFV_6 = a, Age, Genotype, Sex, NRTI, VL_0, Weight_0) \\
& P(Weight_6, MEMS_6, Age, Genotype, Sex, NRTI, VL_0, Weight_0)
\end{aligned} \tag{4}
$$

This is because both dose and co-morbidities are not necessarily needed to block the relevant back-door paths from EFV_t to VL_{t^*}, $t^* \geq t$, i.e. those back-door paths that do not go through any future concentrations.

Note that the identified expressions for θ_{36} and θ_{84} are identical under G_{main} and G_{alt1} because the c-DAG is identical in both situations. Under assumption of G_{alt2}, the c-DAG changes by addition of the variable BHV, resulting in a slight change of the identified expression (see Appendix B.2).

In the second step, we need to decide on the recoverability of the identified expression in terms of the observed data. Note that the missingness mechanism depicted in G_{main} refers to the *closed missingness mechanism* introduced in the previous section. In this case, there is no path between any variable from the c-DAG containing the variables of interest, and the set of missingness indicators M and their causes. Therefore, both causal effects are recoverable, and an available case analysis is admissible in this situation. In order to provide a better intuition about how to decide on the recoverability of a causal effect, we present the recoverability result for θ_{36} in Appendix B.1, implicitly referring to a situation for which no recoverability result exists, and it has to be decided on recoverability manually.

If the m-DAG G_{main} reflects relationships in the data correctly, then available case analysis is sufficient for causal effects of interest to be estimated consistently. However, it is also necessary to assess the plausibility of the graph structure assumption. A suggestion would be to perform sensitivity analysis in terms of investigation of recoverability of the causal effects θ_{36} and θ_{84} under assumption of an alternative m-DAG which is eligible in terms of content. Therefore, we consider another m-DAG, G_{alt1}, which is depicted in Figure 3 when including the dashed blue lines from VL_t to MV_t, $t \in \{0, 6, 36, 48, 60, 84\}$. The arrows from VL_t (binary variable *elevated viral load*) to MV_t (binary variable *missed visit*) for the respective measurement time point reflect the speculation that children with viral failure may miss their appointments due to their poor health condition.

We first turn to investigation of recoverability of θ_{36} under the assumption of G_{alt1} being the true underlying m-DAG. Note that if at least one of the conditional distributions in Equation 3 is non-recoverable, we conclude the non-recoverability of the causal query of interest.

We first focus on the second last conditional distribution in Equation 3 which involves the partially observed data, $P(VL_6|EFV_6 = a, Age, Genotype, Sex, NRTI, VL_0, Weight_0)$, and the goal is to condition on $M_{Weight_0} = 0$, $M_{VL_0} = 0$, $M_{EFV_6} = 0$ and $M_{VL_6} = 0$. From the m-DAG (under assumption of faithfulness), we know that $VL_6 \perp\!\!\!\perp (M_{Weight_0}, M_{VL_0}, M_{EFV_6}, M_{VL_6})|(MV_0, MV_6)$ (and we always need to condition on

(at least) MV_0 and MV_6 to get the (conditional) independency), which in turn leads to

$$
\begin{aligned}
&P(VL_6^{M=0}|EFV_6^{M=0} = a, Age, Genotype, Sex, NRTI, VL_0^{M=0}, Weight_0^{M=0})\\
&= \sum_{MV_0, MV_6} P(VL_6^{M=0}|EFV_6^{M=0} = a, Age, Genotype, Sex, NRTI, VL_0^{M=0}, Weight_0^{M=0})\times\\
&P(MV_0, MV_6|EFV_6^{M=0} = a, Age, Genotype, Sex, NRTI, VL_0^{M=0}, Weight_0^{M=0})\\
&= \sum_{MV_0, MV_6} P(VL_6^{M=0}|EFV_6^{M=0} = a, Age, Genotype, Sex, NRTI, VL_0^{M=0}, Weight_0^{M=0},\\
&M_{Weight_0} = 0, M_{VL_0} = 0, M_{EFV_6} = 0, M_{VL_6} = 0)\times\\
&P(MV_0, MV_6|EFV_6 = a^{M=0}, Age, Genotype, Sex, NRTI, VL_0^{M=0}, Weight_0^{M=0})\\
&= \sum_{MV_0, MV_6} P(VL_6|EFV_6 = a, Age, Genotype, Sex, NRTI, VL_0, Weight_0,\\
&M_{Weight_0} = 0, M_{VL_0} = 0, M_{EFV_6} = 0, M_{VL_6} = 0)\times\\
&P(MV_0, MV_6|EFV_6 = a^{M=0}, Age, Genotype, Sex, NRTI, VL_0^{M=0}, Weight_0^{M=0}).
\end{aligned}
$$

The second factor in the expression above,

$$P(MV_0, MV_6|EFV_6^{M=0} = a, Age, Genotype, Sex, NRTI, VL_0^{M=0}, Weight_0^{M=0}),$$

cannot be further decomposed following the adjustment formula, as among others we would need MV_6 to be conditionally independent of M_{EFV_6} (possibly given some other variables), but there is no such subset because MV_6 is a parent of M_{EFV_6}.

Because other available recoverability techniques, like sequential factorization or R factorization (Mohan and Pearl, 2021), are not applicable in our case, we conclude that the conditional distribution cannot be expressed in terms of the observed data distribution only; this results in non-recoverability of θ_{36}. Based on the same arguments, non-recoverability of θ_{84} follows.

Analysis

The analysis is conducted using the m-DAG G_{main} from Figure 3 and the study data described in Section 4.1. Plug-in g-formula estimation (see, e.g., Hernan and Robins (Hernán and Robins, 2020, Chapter 13)) of our estimands 1 and 2 was based on equations 4 and 6, respectively. Provided the recoverability under G_{main}, we conduct the analysis based on available cases. For modeling of the respective conditional distributions, between 69 and 85 complete observations are available.

Note that in our setting, relevant co-morbidities for the paths represented in the DAG refer to AIDS defining events, which include tuberculosis, persistent diarrhea, malnutrition, severe wasting, among others. Most of those events were not measured regularly in our study and were thus not included in the analysis. However, those co-morbidities are not expected to be very frequent in the study population anyway. This is because children with active infections, being treated for tuberculosis and with severe laboratory abnormalities at screening, were not enrolled into the study, and most children were in relatively good health (Mulenga et al., 2016). Also, not all valid identification formulae require information on them, e.g. those used in our analysis. As adherence could not be measured regularly, as explained in Section 4.1, we decided to construct a variable which indicated whether there was any sign of non-adherence, defined as the mean memory caps opening percentage being smaller than 90%.

While the main analysis was based on an available case analysis, owing to the identification results, we decided to also implement a multiple imputation analysis. We consider the results of the multiple imputation analysis not to be valid because the underlying missingness mechanism is assumed to be MNAR. This is predominantly due to the unobserved variables (TI_t), which are directly causing missingness in variables from the c-DAG.

We multiply imputed 5 data sets using the Expectation-Maximization Bootstrap (EMB) Algorithm, which is a joint modeling based imputation approach implemented in the *R*-package `Amelia II`. Our setup considered the longitudinal setup through lag-variables and the inclusion of splines of time. Additionally, to improve numerical stability of the EM algorithm, we added a ridge prior (which shrinks the covariances among the variables toward zero without changing the means or variances). This is often recommended when using EMB on small samples (Honaker et al., 2011). To address that age, weight and EFV concentrations can not have negative or other illogical values, we specified lower and upper bounds for those variables in the EMB algorithm,

i.e. 0 and 35 for EFV, 0 and 3 for log age and 2 and 4 for log weight. `Amelia II` implements these bounds by rejection sampling: when drawing the imputations from the respective posterior distributions, all logical constraints need to be satisfied, otherwise imputations are redrawn until those are met.

The results of our analyses are presented in Figures 4 and 5.

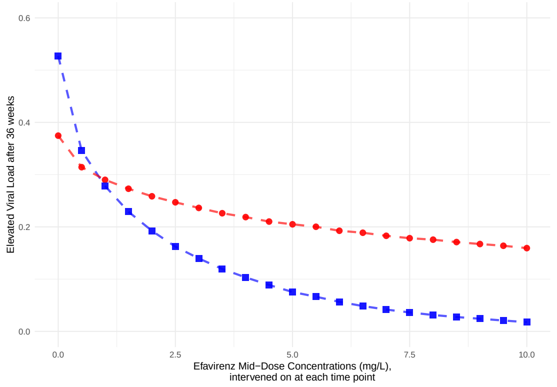

Fig. 4. Estimated CCRCs for θ_{36} under m-DAG G_{main}; causal effects estimated using available cases (blue squares) and multiple imputation, mean over 20 imputed data sets (red dots); mean over 1000 seeds

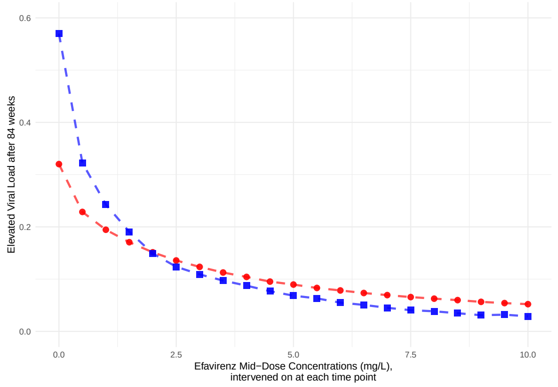

Fig. 5. Estimated CCRCs for θ_{84} under m-DAG G_{main}; causal effects estimated using available cases (blue squares) and multiple imputation (red dots), mean over 20 imputed data sets; mean over 1000 seeds

One can see a higher probability of viral failure with lower EFV concentration values, independent of the approach employed to address the missing data. Using the suggested available cases approach, the probability of failure is estimated to be $> 50\%$ at both 36 and 84 weeks if concentrations were 0mg/L, e.g. if patients did not take any medications. With higher concentrations, failure probabilities reduce to below 5%, which is expected as EFV is expected to be a potent drug. Interestingly, the CCRCs which were estimated using multiple imputation are much flatter than under the available case approach, both for θ_{36} and θ_{84}.

We will now explore the derived theoretical results, as well as an available case and MI comparisons in simulation studies below.

SIMULATION STUDIES

In this section, through simulation studies, we assess the reliability of CCRC estimates for θ_{84} in the presence of missing data. We compare true CCRC values with estimates derived from: (i) an ideal scenario with no missing data (complete data analysis), (ii) available case analysis, and (iii) multiple imputation.

Setup

The data was simulated with help of the `R`-package `simcausal` (Sofrygin et al., 2017), which provides a powerful and flexible tool for simulation of longitudinal data structures based on structural causal models. A structural causal model (SCM) uniquely imposes a causal graph, which means that data structures can be generated according to such a causal DAG (in our case, an m-DAG).

Simulation 1: We separately simulate the data corresponding to both m-DAGs, G_{main} and G_{alt1}, as defined in Figure 3. We first simulate binary and normally distributed confounder sets, a continuous (truncated normally distributed) intervention and a binary outcome for all 6 time points and for the sample size of $n = 5.000$. This way, the data aligning with the c-DAG is simulated. Afterwards, we simulate the binary variables causing missingness (SES_t (as proxy for BMQ_t, SES_t and BHV_t), MV_t and TI_t, $t \in \{0, 6, 36, 48, 60, 84\}$. Based on these variables, we simulate the missingness indicators for VL_t, $t \in \{0, 6, 36, 48, 60, 84\}$, and $MEMS_t$, $t \in \{6, 36, 48, 60, 84\}$. In this case, we assume that only these two variables (for all time points) are subject to missing data.

Simulation 2: The DGP is the same as for simulation 1, but we simulate missingness indicators for much more variables: VL_t, $Weight_t$, $t \in \{0, 6, 36, 48, 60, 84\}$, and $MEMS_t$, EFV_t, $t \in \{6, 36, 48, 60, 84\}$. This mostly coincides with the GPD induced by Figure 3. Note that this scenario introduces another layer of complexity because the distribution of EFV_t, $t \in \{6, 36, 48, 60, 84\}$, is non-symmetric and complex; which means that using a parametric imputation model with slightly misspecified distributional assumptions may introduce some bias. This setting serves as a benchmark for the realistic scenario where missing data of somewhat complex distributions are imputed using parametric assumptions in conjunction with predictive mean matching (fully conditional imputation procedures, like (M)ICE), or variations thereof (joint modeling, as in `Amelia II` which we use below).

The exact model specifications are given in Appendix C.1. Based on the DGP, we first simulate the interventional data (1000 repetitions), and compute the true CCRC based on it. We intervene on EFV at time points $6, 36, 48, 60$, and 84, considering a sequence of interventions of interest from 0 up to 10 mg/L with steps of 0.5 mg/L. Next, the CCRC is estimated using the g-formula factorization as provided in Appendix A.1, once on the complete data and once on the available case data. Compared to the widely used complete case analysis, which relies on samples in which variables in the entire dataset are observed, the available case analysis retains all samples in which variables in the query of interest (for the g-formula, the variables required for the estimation of the conditional distribution of interest) are observed. Because of this, an available case analysis is preferred over a complete case analysis due to a more economical usage of available data (Mohan and Pearl, 2021). Finally, the CCRC is also estimated using multiple imputation (MI) and the g-formula.

Results

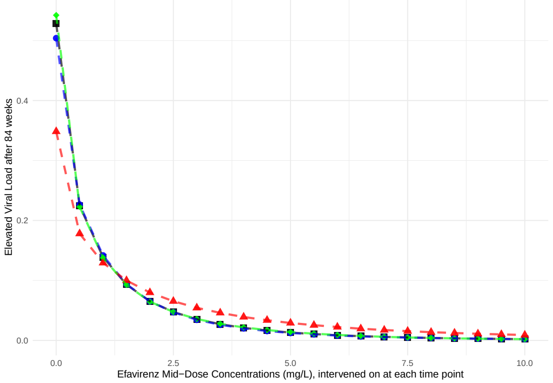

Fig. 6. Estimated CCRCs for probability of viral failure after 84 weeks based on data simulated under the main DAG G_{main}; causal effects estimated on complete (black squares), incomplete data using available cases (blue dots), incomplete data using multiple imputation (red triangles) and counterfactual (green diamonds, true CCRC) data; mean over 1000 seeds

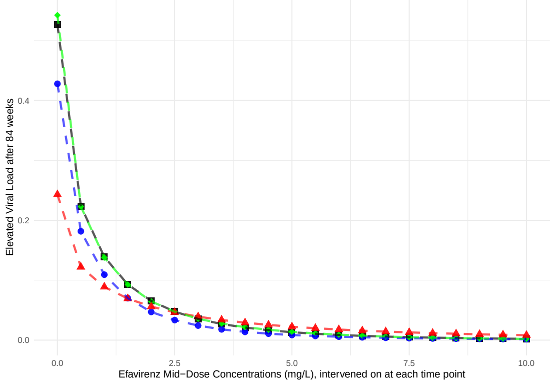

Fig. 7. Estimated CCRCs for probability of viral failure after 84 weeks based on data simulated under the alternative DAG G_{alt1}; causal effects estimated on complete (black squares), incomplete data using available cases (blue dots), incomplete data using multiple imputation (red triangles) and counterfactual (green diamonds, true CCRC) data; mean over 1000 seeds

Below, we focus on the results from simulation 1. The results of the second simulation are presented in Appendix C.2, i.e. in Figures 8 and 9.

The simulation results are in accordance with the theoretical findings. We first consider the estimated CCRC curve for elevated viral load after 84 weeks based on data simulated under G_{main}, presented in Figure 6. According to the findings in Section 4, the missingness mechanism is of MNAR type. Despite this, the causal query of interest, θ_{84}, remains recoverable, allowing for consistent estimation of the CCRC via an available case analysis. Conversely, the MI approach is inadmissible due to its underlying assumption of Missing At Random (MAR), which is not met because of the partially observed variables TI_t, $t \in 0, 6, 36, 48, 60, 84$, that affect the probability of missingness in variables from V_m. The estimated counterfactual outcomes, illustrated in Figure

6, represent the mean estimated values across 1.000 simulation repetitions. It is evident that the CCRC estimates from available case analysis (dashed blue line) match the true CCRC (dashed green line), whereas those derived from MI differ markedly. To explore whether the latter discrepancies present a real bias or could also be explained by simulation uncertainty, we calculated the Monte Carlo confidence intervals for the differences between the MI estimation results and the true causal effects θ_{84}. The results are reported in Table 1 and show that for each EFV value the estimated differences are markedly different from zero. This discrepancy underscores the theoretical findings about the bias introduced by MI due to the MNAR missingness mechanism.

Now, secondly, consider the results under the alternative DAG G_{alt1}, presented in Figure 7. Here, the causal query of interest is non-recoverable. The results confirm this and demonstrate that neither the available case analysis (dashed blue line) nor the MI estimates (dashed red line) align with the true CCRC (dashed green line). The respective Monte Carlo confidence intervals, see Tables 2 and 3 in the appendix, show that those differences cannot be explained by simulation uncertainty alone – which is in line with the theoretical findings.

These findings highlight the critical need to scrutinize the common MAR-type missingness assumption, especially in complex longitudinal data scenarios with multiple variables experiencing missingness. Our simulation study illustrates how even minor changes in the missingness structure can dramatically affect the recoverability and, consequently, the accuracy of estimation results. This emphasizes the importance of careful consideration and justification of missingness assumptions in such analyses.

CONCLUSIONS

Our analyses show the applicability of missingness DAGs to complex longitudinal studies, and that sometimes available case analyses can be valid under MNAR. However, our application also highlights the massive effort involved, technical expertise required and sensitivity of results with respect to the assumed causal model.

Under the assumptions represented in Figure 3, the data are missing not at random (MNAR) because technical issues (TI) with pill containers (frequently) and blood samples (rarely), which we assume to be direct causes of missingness in multiple variables, have not been measured. However, we show that the assumptions are sufficient to estimate the desired effect by means of the available data, using g-formula representations – despite MNAR. If reasons for missed visits are caused by the outcome (elevated viral load), as speculated in the alternative DAG, the causal effect can however not be recovered. Interestingly, additional simulations (Appendix C.2, Figure 10) show that recoverability holds true even if behavioural factors directly cause adherence. This corresponds to the situation discussed in the second alternative DAG. It is however possible that there are more complex processes between socio-economic/behavioral factors and biologic processes for which identifiability does not hold.

Additionally, it may be important to highlight that the estimated concentration-response curves are much flatter when using multiple imputation, and are actually invalid under the assumptions stated in Figure 3 – as predicted by theory and confirmed by us in simulations.

Our analyses show lower probabilities of viral failure with higher concentrations, after taking into account the problems of missing data. There are, of course, many further complications that may have to be considered for accurate causal inference in our setting. First, one may also account for measurement error, e.g. in viral load and EFV concentrations (Schomaker et al., 2015), though this may not affect our binary viral failure definition very much. Second, it would be advantageous if measurements would be available even more frequently and precisely, specifically for measuring actual adherence to the prescribed treatment plan, which is difficult in practice.

While the advancements in causal modeling and appropriate statistical estimation techniques are impressive, answering complex epidemiological and biological questions remains a challenge.

IDENTIFIABILITY RESULTS

Identifiability results for θ_{84}

The identifiability result below is based on application of the IDC algorithm (Shpitser and Pearl, 2006).

$$
\begin{aligned}
\theta_{84} = \sum_{\substack{Age,Sex,CoMo_0,Weight_0,\\ MEMS_6,Weight_6,VL_0,CoMo_6,\\ VL_6,MEMS_{36},Weight_{36},CoMo_{36},\\ MEMS_{48},Weight_{48},VL_{36},CoMo_{48},\\ VL_{48},CoMo_{60},VL_{60}}} & P(VL_{84} = 1 | Age, Sex, CoMo_0, Weight_0, Genotype, MEMS_6, Weight_6, \\
& Dose_6, EFV_6 = a, VL_0, CoMo_6, VL_6, MEMS_{36}, \\
& Weight_{36}, CoMo_{36}, Dose_{36}, MEMS_{48}, Weight_{48}, EFV_{36} = a, \\
& Dose_{48}, VL_{36}, EFV_{48} = a, CoMo_{48}, VL_{48}, MEMS_{60}, Weight_{60}, CoMo_{60}, \\
& Dose_{60}, MEMS_{84}, Weight_{84}, EFV_{60} = a, Dose_{84}, VL_{60}, EFV_{84} = a) \\
& P(VL_{60} | Age, Sex, CoMo_0, Weight_0, Genotype, MEMS_6, Weight_6, \\
& Dose_6, EFV_6 = a, VL_0, CoMo_6, VL_6, MEMS_{36}, \\
& Weight_{36}, CoMo_{36}, Dose_{36}, MEMS_{48}, Weight_{48}, EFV_{36} = a, Dose_{48}, \\
& VL_{36}, EFV_{48} = a, CoMo_{48}, VL_{48}, MEMS_{60}, Weight_{60}, Dose_{60}, EFV_{60} = a) \\
& P(CoMo_{60} | Age, Sex, CoMo_0, Weight_0, Genotype, MEMS_6, Weight_6, \\
& Dose_{36}, Dose_6, EFV_6 = a, VL_0, CoMo_6, VL_6, MEMS_{36}, Weight_{36}, CoMo_{36}, \\
& MEMS_{48}, Weight_{48}, EFV_{36} = a, Dose_{48}, VL_{36}, EFV_{48} = a, CoMo_{48}, VL_{48}) \\
& P(VL_{48} | Age, Sex, CoMo_0, Weight_0, Genotype, MEMS_6, Weight_6, Dose_6, \\
& EFV_6 = a, VL_0, CoMo_6, VL_6, MEMS_{36}, Weight_{36}, CoMo_{36}, Dose_{36}, \\
& MEMS_{48}, Weight_{48}, EFV_{36} = a, Dose_{48}, VL_{36}, EFV_{48} = a) \\
& P(CoMo_{48} | Age, Sex, CoMo_0, Weight_0, Genotype, MEMS_6, Weight_6, \qquad (5) \\
& Dose_6, EFV_6 = a, VL_0, CoMo_6, VL_6, MEMS_{36}, \\
& Weight_{36}, CoMo_{36}, Dose_{36}, EFV_{36} = a, VL_{36}) \\
& P(Weight_{48} | Age, Sex, CoMo_0, Weight_0, Genotype, MEMS_6, Weight_6, \\
& Dose_6, EFV_6 = a, VL_0, CoMo_6, VL_6, Weight_{36}, CoMo_{36}) \\
& P(MEMS_{48} | Age, Sex, CoMo_0, Weight_0, Genotype, MEMS_6, Weight_6, \\
& Dose_6, EFV_6 = a, VL_0, CoMo_6, VL_6, MEMS_{36}, CoMo_{36}) \\
& P(CoMo_{36} | Age, Sex, CoMo_0, Weight_0, Genotype, MEMS_6, Weight_6, \\
& Dose_6, EFV_6 = a, VL_0, CoMo_6, VL_6) \\
& P(VL_{36} | Age, Sex, CoMo_0, Weight_0, Genotype, MEMS_6, Weight_6, Dose_6, \\
& EFV_6 = a, VL_0, CoMo_6, VL_6, MEMS_{36}, Weight_{36}, Dose_{36}, EFV_{36} = a) \\
& P(Weight_{36} | Age, Sex, CoMo_0, Weight_0, Genotype, Weight_6, \\
& VL_0, CoMo_6) P(MEMS_{36} | Age, Sex, CoMo_0, Weight_0, Genotype, MEMS_6, \\
& VL_0, CoMo_6) P(VL_6 | Age, Sex, CoMo_0, Weight_0, Genotype, \\
& MEMS_6, Weight_6, Dose_6, EFV_6 = a, VL_0) \\
& P(CoMo_6 | Age, Sex, CoMo_0, Weight_0, Genotype, VL_0) \\
& P(Weight_6 | Age, Sex, CoMo_0, Weight_0) P(MEMS_6 | CoMo_0) \\
& P(Weight_0 | Age, Sex) P(CoMo_0 | Age, Sex) P(VL_0) P(Sex) P(Age)
\end{aligned}
$$

An alternative identifiability result can be obtained, for example, by applying the generalized back-door criterion. This leads to using an adjustment set given by the confounders weight and adherence (MEMS). Both dose and co-morbidities are not necessarily needed to block the relevant back-door paths from EFV_t to VL_{t^*}, $t^* \geq t$, i.e. those back-door paths that do not go through any future concentrations (Hernán and Robins, 2020). Applying Robins' parametric g-formula (Robins, 1986; Hernán and Robins, 2020), then leads to the following valid g-formula factorization:

$$\theta_{84} = \int_l \Bigg\{ P(VL_{84} = 1 | \bar{A}_{84} = \bar{a}_{84}, \bar{\mathbf{L}}_{84} = \bar{\mathbf{l}}_{84}) \times \prod_{s=0}^{84} f(\mathbf{L}_s = \mathbf{l}_s | \bar{A}_{s-1} = \bar{a}_{s-1}, \bar{\mathbf{L}}_{s-1} = \bar{\mathbf{l}}_{s-1}) \Bigg\} \, dF_l(l) \,, \tag{6}$$

where $F_L(\cdot)$ denotes the CDF with respect to L. In the above, $\mathbf{L}_t = \{\text{weight}_t, \text{MEMS}_t\}$, $\mathbf{L}_0 = \{\text{weight}_0, \text{NRTI}, \text{Genotype}, \text{Sex}, \text{Age}\}$ and $A_t = \text{EFV}_t$. Note that the overbar refers to the intervention and confounder histories, i.e. for of a unit i (up to and including time t) the histories are$\bar{A}_{t,i} = (A_{0,i}, \ldots, A_{t,i})$ and $\bar{L}^s_{t,i} = (L^s_{0,i}, \ldots, L^s_{t,i})$, $s = 1, 2$, $i = 1, \ldots, n$, respectively. Outcomes (i.e., VL) prior to s are part of $\bar{\mathbf{L}}_s$. The inner product of the 2 time-varying and ordered confounders $\mathbf{L}_s = \{L^1_s, L^2_s\}$ can be decomposed further:

$$\prod_{s=0}^{84} f(L^2_s = l^2_s \mid L^1_s = l^1_s, \bar{A}_{s-1} = \bar{a}_{s-1}, \bar{L}_{s-1} = \bar{\mathbf{l}}_{s-1}) \times f(L^1_s = l^1_s \mid \bar{A}_{s-1} = \bar{a}_{s-1}, \bar{\mathbf{L}}_{s-1} = \bar{\mathbf{l}}_{s-1})$$

Thus, implementing (6), requires fitting models for the outcome and two confounder distributions at each time point, given their respective history.

RECOVERABILITY RESULTS FOR θ_{36}

Assuming $\mathbf{G_{main}}$

In order to assess the recoverability of the identified causal effect θ_{36} in Equation 3, we have to show that each of the multiplicative factors corresponding to the conditional or marginal distributions can be expressed in terms of the observed data only. Despite the fact that the missingness mechanism is of type 'closed', and the recoverability result is directly available for this case, we still want to demonstrate how one would proceed in the case in which such a result is not available. For doing so, we present the result for one of the conditional distributions containing partially observed variables. The results for other multiplicative factors are derived analogously.

$$\begin{aligned}
&P(CoMo_6^{M=0} | Age, Sex, CoMo_0^{M=0}, Weight_0^{M=0}, Genotype, VL_0^{M=0}) \\
=&P(CoMo_6^{M=0} | Age, Sex, CoMo_0^{M=0}, Weight_0^{M=0}, Genotype, VL_0^{M=0}, \mathbf{M} = 0) \\
=&P(CoMo_6 | Age, Sex, CoMo_0, Weight_0, Genotype, VL_0, \mathbf{M} = 0)
\end{aligned}$$

The first equality holds due to the fact that all partially observed variables are independent of the corresponding relevant missingness indicators, whereas the second equality is true due to the consistency assumption (Moreno-Betancur et al., 2018).

Note that the recoverability result above is very much trivial due to the specific missingness mechanism. In a general case, much more effort has to be put in the attempt to recover a distribution of interest.

Assuming $\mathbf{G_{alt2}}$

We consider a second plausible alternative m-DAG, G_{alt2}, which is equivalent to G_{main} in Figure 3 with addition of a direct effect of BHV on $MEMS$ for weeks 6, 36, 48, 60 and 84. In this way, we argue that behavioural pattern may affect adherence, which is measured through memory caps.

Under G_{alt2}, we get the following identified expression for the causal effect of interest, θ_{36}:

$$\begin{aligned}\theta_{36} = \sum_{\substack{Age,Sex,CoMo_0,Weight_0,\\ VL_0,CoMo_6,VL_6}} & P(VL_{36}|Age, Sex, BHV, CoMo_0, Weight_0, Genotype, MEMS_6,\\ & Weight_6, Dose_6, EFV_6 = a, VL_0, CoMo_6, VL_6, MEMS_{36}, Weight_{36},\\ & Dose_{36}, EFV_{36} = a)\\ & P(VL_6|Age, Sex, CoMo_0, Weight_0, Genotype, MEMS_6,\\ & Weight_6, Dose_6, EFV_6 = a, VL_0)\\ & P(CoMo_6|Age, Sex, CoMo_0, Weight_0, Genotype, VL_0)\\ & P(Weight_0|Age, Sex)P(CoMo_0|Age, Sex)P(VL_0)P(Sex)P(Age).\end{aligned} \tag{7}$$

Note that even if the identified expression look almost identical to the one we get under G_{main} or G_{alt1} (compare Equation 3), the recoverability result may be different due to the different conditional independence statements which hold in G_{alt2} assumed it is faithful.

SIMULATION STUDIES

DGP for Simulations 1 and 2

Both baseline data ($t = 0$) and follow-up data ($t = 1, \dots, 5$) were created using structural equations using the R-package `simcausal`. Note that the follow-up time points 1 up to 5 correspond to the study weeks 6, 36, 48, 60 and 84. The below listed distributions, listed in temporal order, describe the data-generating process. Our baseline data consists of *Sex*, *Genotype*, log(age) (*Age*), log(weight) (*Weight*), the respective Nucleoside Reverse Transcriptase Inhibitor (*NRTI*), and the proxy for socio-economic status (SES). Time-varying variables are co-morbidities (CoMo), efavirenz dose (Dose), efavirenz mid-dose concentration (EFV), elevated viral load (= viral failure, VL), adherence (measured through memory caps, MEMS), missed visit (MV), technical issue (TI), and the missingness indicators for EFV, $Weight$, VL, and $MEMS$, respectively. In addition to Bernoulli (B), Poisson ($Poisson$), Multinominal (MN) and Normal (N) distributions, we also use truncated normal distributions; they are denoted by $N_{[a,a_1,a_2,b,b_1,b_2]}$, where a and b are the truncation levels. Values which are smaller than a are replaced by a random draw from a $U(a_1, a_2)$ distribution and values greater than b are drawn from a $U(b_1, b_2)$ distribution, where U refers to a continuous uniform distribution. For the specified multinomial distributions, probabilities are normalized, if required, such that they add up to 1.

The DGP corresponding to G_{main} in Figure 3 is as following:

For $t = 0$:

$$\begin{aligned}
\mathrm{Sex}_0 &\sim \mathrm{B}(p = 0.5)\\
\mathrm{Genotype}_0 &\sim \mathrm{MN}\begin{pmatrix} p1 = 1/(1+\exp(-(-0.103 + \mathrm{I}(\mathrm{Sex}_0 = 1)\times 0.223 + \mathrm{I}(\mathrm{Sex}_0 = 0)\times 0.173))),\\ p2 = 1/(1+\exp(-(-0.086 + \mathrm{I}(\mathrm{Sex}_0 = 1)\times 0.198 + \mathrm{I}(\mathrm{Sex}_0 = 0)\times 0.214))),\\ p3 = 1/(1+\exp(-(-0.309 + \mathrm{I}(\mathrm{Sex}_0 = 1)\times 0.082 + \mathrm{I}(\mathrm{Sex}_0 = 0)\times 0.170)))\end{pmatrix}\\
\mathrm{Age}_0 &\sim N_{[0.693,0.693,1,2.8,2.7,2.8]}(\mu = 1.501, \sigma = 0.369)\\
\mathrm{Weight}_0 &\sim N_{[2.26,2.26,2.67,3.37,3.02,3.37]}(\mu = (1.5 + 0.2\times \mathrm{Sex}_0 + 0.774\times \mathrm{Age}_0)\times 0.94), \sigma = 0.369)\\
\mathrm{NRTI}_0 &\sim \mathrm{MN}\begin{pmatrix} p1 = 1/(1+\exp(-(-0.006 + \mathrm{I}(\mathrm{Age}_0 > 1.4563)\times \mathrm{Age}_0\times 0.1735 + \mathrm{I}(\mathrm{Age}_0 \le 1.4563)\times \mathrm{Age}_0\times 0.1570))),\\ p2 = 1/(1+\exp(-(-0.006 + \mathrm{I}(\mathrm{Age}_0 > 1.4563)\times \mathrm{Age}_0\times 0.1735 + \mathrm{I}(\mathrm{Age}_0 \le 1.4563)\times \mathrm{Age}_0\times 0.1570))),\\ p3 = 1/(1+\exp(-(-0.006 + \mathrm{I}(\mathrm{Age}_0 > 1.4563)\times \mathrm{Age}_0\times 0.1570 + \mathrm{I}(\mathrm{Age}_0 \le .14563)\times \mathrm{Age}_0\times 0.1818)))\end{pmatrix}\\
\mathrm{CoMo}_0 &\sim \mathrm{B}(p = 0.15)\\
\mathrm{VL}_0 &\sim \mathrm{B}(p = 1 - (1/(1+\exp(-(0.4 + 1.9\times \sqrt{\mathrm{EFV}_0})))))\\
\mathrm{SES}_0 &\sim Poisson(\lambda = 3)\\
\mathrm{MV}_0 &\sim \mathrm{B}(p = 1/(1+\exp(-(-2.95 + 0.1\times \mathrm{SES}_0))))
\end{aligned}$$

For $t = 1$:

$$\text{Dose}_1 \sim \text{MN}\begin{pmatrix} p1 = 1/(1+\exp(-(5+\sqrt{(\text{Weight}_0)}\times 8 - \text{Age}_0 \times 10)))\,, \\ p2 = 1/(1+\exp(-(4+\sqrt{(\text{Weight}_0)}\times 8.768 - \text{Age}_0 \times 9.06)))\,, \\ p3 = 1/(1+\exp(-(3+\sqrt{(\text{Weight}_0)}\times 6.562 - \text{Age}_0 \times 8.325)))\,, \\ p4 = 1-(p1+p2+p3) \end{pmatrix}$$

For $t \geq 0$:

$$\begin{aligned} \text{TI}_t &\sim \text{B}(p = 0.05)))) \\ \text{M}_{EFV_t} &\sim \text{B}(p = I(\text{MV}_t = 1) + I(\text{MV}_t = 0) \times I(\text{TI}_t = 1) \times 0.5)))) \\ \text{M}_{Weight_t} &\sim \text{B}(p = I(\text{MV}_t = 1)) \\ \text{M}_{VL_t} &\sim \text{B}(p = I(\text{MV}_t = 1) + I(\text{MV}_t = 0) \times I(\text{TI}_t = 1) \times 0.5)))) \end{aligned}$$

For $t \geq 1$:

$$\begin{aligned} \text{MEMS}_t &\sim \text{B}(p = 1/(1+\exp(-(0.71 + \text{CoMo}_{t-1} \times 0.31 + \text{MEMS}_{t-1} \times \text{I}(t \geq 2) \times 0.31)))), \quad [\text{assume MEMS}_0 = 0] \\ \text{Weight}_t &\sim N_{[2.26,2.26,2.473,3.37,3.2,3.37]}(\mu = \text{Weight}_{t-1} \times 1.04 - 0.05 \times \text{I}(\text{CoMo}_{t-1} = 1),\ \sigma = 0.4) \\ \text{CoMo}_t &\sim \text{B}(p = 1 - (1/(1+\exp(-(0.5 \times \text{I}(\text{CoMo}_{t-1} = 1) + \text{Age}_0 \times 0.1 + \text{Weight}_{t-1} \times 0.1))))) \\ \text{EFV}_t &\sim N_{[0.2032,0.2032,0.88,21.84,8.37,21.84]}(\mu = 0.1 \times \text{Dose}_t + 0.1 \times \text{MEMS}_t + \text{I}(\text{Genotype}_0 \leq 2) \times 2.66 \\ &\quad + \text{I}(\text{Genotype}_0 = 3) \times 4.6, \sigma = 4.06) \\ \text{VL}_t &\sim \text{B}(p = 1 - (1/(1+\exp(-(1 - 0.6 \times \text{I}(t=1) - 1.2 \times \text{I}(t=4) + 0.1 \times \text{CoMo}_{t-1} + (2 - 0.2 \times \text{I}(t=3)) \times \sqrt{\text{EFV}_t}))))) \\ \text{MV}_t &\sim \text{B}(p = 1/(1+\exp(-(-2.95 + 0.1 \times \text{SES}_0 + \text{MV}_{t-1})))) \\ \text{M}_{MEMS_t} &\sim \text{B}(p = 1/(1+\exp(-(0.5 \times I(\text{TI}_t = 1) + 0.2)))) \end{aligned}$$

For $t \geq 2$:

$$\text{Dose}_t \sim \text{MN}\begin{pmatrix} p1 = (1/(1+\exp(-(4+\text{Dose}_{t-1} \times 0.5 + \sqrt{\text{Weight}_t} \times 4 - \text{Age}_0 \times 10)))\,, \\ p2 = (1/(1+\exp(-(-8+\text{Dose}_{t-1} \times 0.5 + \sqrt{\text{Weight}_t} \times 8.568 - \text{Age}_0 \times 9.06)))\,, \\ p3 = (1/(1+\exp(-(20+\text{Dose}_{t-1} \times 0.5 + \sqrt{\text{Weight}_t} \times 6.562 - \text{Age}_0 \times 18.325)))\,, \\ p4 = 1-(p1+p2+p3) \end{pmatrix}$$

The DGP for G_{alt1} (with present blue dashed lines in Figure 3) coincides with the DGP above, except of the structural equations for MV_t, $t \in \{0, 6, 36, 48, 60, 84\}$. These are specified as following for G_{alt1}:

For $t = 0$:

$$\text{MV}_0 \sim \text{B}(p = 1/(1+\exp(-(-2.95 + 0.1 \times \text{SES}_0 + 2 \times \text{VL}_0))))$$

For $t \geq 1$:

$$\text{MV}_t \sim \text{B}(p = 1/(1+\exp(-(-2.95 + 0.1 \times \text{SES}_0 + \text{MV}_{t-1} + 2 \times \text{VL}_t))))$$

This way, MV_t additionally depends on VL_t, $t \in \{0, 6, 36, 48, 60, 84\}$, which corresponds to the dashed blue lines in the DAG.

The DGP for G_{alt2} (with SES being a cause of $MEMS_t$, $t \in \{6, 36, 48, 60, 84\}$) coincides with the DGP for G_{main} above, except of the structural equations for $MEMS_t$, $t \in \{6, 36, 48, 60, 84\}$. These are specified

for G_{alt2} as following:
For $t \geq 1$:

$$\text{MEMS}_t \sim \text{B}(p = 1/(1 + \exp(-(0.71 + \text{CoMo}_{t-1} \times 0.31 + \text{MEMS}_{t-1} \times \text{I}(t \geq 2) \times 0.31 - \text{SES}_0 \times 0.5))))$$

After a data set has been generated using the structural equations, we introduce the missing values corresponding to the missingness indicators: if the value of a missingness indicator equals 1, the corresponding covariate value is set to `NA`. In *Simulation 1*, we ignore the missingness indicators for EFV_t, $t \in \{6, 36, 48, 60, 84\}$, and $Weight_t$, $t \in \{0, 6, 36, 48, 60, 84\}$, and only generate missingness in VL_t and $MEMS_t$, $t \in \{0, 6, 36, 48, 60, 84\}$. In *Simulation 2*, missingness is introduced in EFV_t, $t \in \{6, 36, 48, 60, 84\}$, $Weight_t$, VL_t and $MEMS_t$, $t \in \{0, 6, 36, 48, 60, 84\}$.

Results

The results below are based on simulation 2 as defined in Section 5.

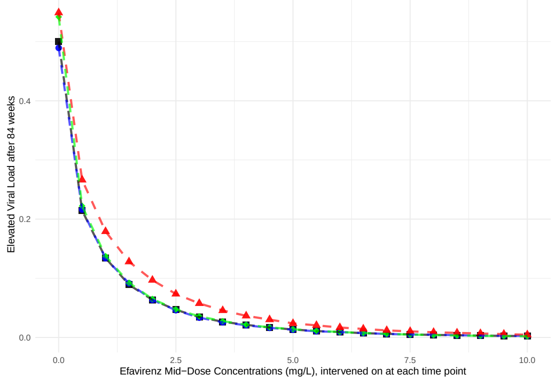

Fig. 8. Estimated CCRCs for probability of viral failure after 84 weeks based on data simulated under the main DAG G_{main}; causal effects estimated on complete (black squares), incomplete data using available cases (blue dots), incomplete data using multiple imputation (red triangles) and counterfactual (green diamonds, true CCRC) data; mean over 1000 seeds

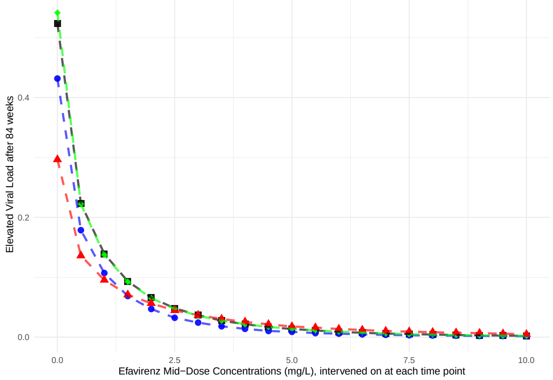

Fig. 9. Estimated CCRCs for probability of viral failure after 84 weeks based on data simulated under the alternative DAG G_{alt1}; causal effects estimated on complete (black squares), incomplete data using available cases (blue dots), incomplete data using multiple imputation (red triangles) and counterfactual (green diamonds, true CCRC) data; mean over 1000 seeds

The following results are based on simulation 1 as defined in Section 5 under the assumption of G_{alt2} being the true underlying causal m-DAG.

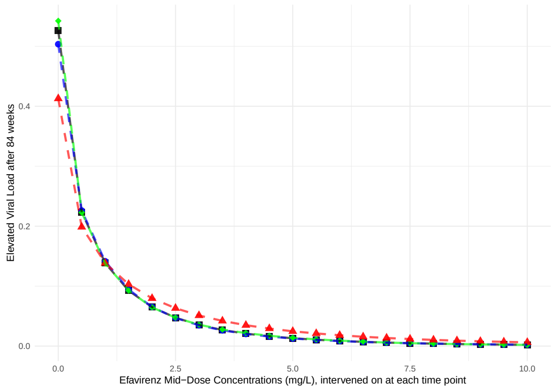

Fig. 10. Estimated CCRCs for probability of viral failure after 84 weeks based on data simulated under the main DAG G_{alt2}; causal effects estimated on complete (black squares), incomplete data using available cases (blue dots), incomplete data using multiple imputation (red triangles) and counterfactual (green diamonds, true CCRC) data; mean over 1000 seeds

Monte Carlo Confidence Intervals

The below Monte Carlo confidence intervals are reported to check whether the differences between MI estimates and true values (main and alternative m-DAGs, G_{main} and G_{alt1}), and between available case analysis estimates and true causal effects θ_{84} (alternative m-DAG G_{alt1}) stem from simulation uncertainty or indicate systematic deviations. A 95% confidence interval (mean difference $\pm$ $2SE$), with the standard error SE computed as $sd(estimate)/\sqrt{(\#runs)}$, excluding zero suggests that the differences can likely not be explained by simulation uncertainty, pointing towards a bias caused by the estimation approach.

EFV	Lower	Upper
0.0	-0.19441	-0.19351
0.5	-0.04522	-0.04452
1.0	-0.00881	-0.00824
1.5	0.00696	0.00749
2.0	0.01493	0.01539
2.5	0.01774	0.01817
3.0	0.01842	0.01880
3.5	0.01812	0.01847
4.0	0.01721	0.01754
4.5	0.01626	0.01656
5.0	0.01502	0.01530
5.5	0.01403	0.01429
6.0	0.01309	0.01335
6.5	0.01191	0.01214
7.0	0.01123	0.01145
7.5	0.01051	0.01071
8.0	0.00952	0.00971
8.5	0.00886	0.00904
9.0	0.00810	0.00827
9.5	0.00750	0.00766
10.0	0.00685	0.00701

Table 1. Monte Carlo confidence intervals for the difference between MI estimates and true causal effects across efavirenz concentrations under G_{main}

EFV	Lower	Upper
0.0	-0.12787	-0.10112
0.5	-0.04754	-0.03497
1.0	-0.03208	-0.02512
1.5	-0.02465	-0.02075
2.0	-0.01873	-0.01642
2.5	-0.01470	-0.01320
3.0	-0.01195	-0.01092
3.5	-0.01012	-0.00935
4.0	-0.00823	-0.00759
4.5	-0.00677	-0.00620
5.0	-0.00558	-0.00509
5.5	-0.00450	-0.00405
6.0	-0.00358	-0.00317
6.5	-0.00296	-0.00257
7.0	-0.00222	-0.00186
7.5	-0.00175	-0.00141
8.0	-0.00145	-0.00115
8.5	-0.00114	-0.00085
9.0	-0.00088	-0.00062
9.5	-0.00074	-0.00048
10.0	-0.00058	-0.00034

Table 2. Monte Carlo confidence intervals for the difference between available case estimates and true causal effects across efavirenz concentrations under G_{alt1}

EFV	Lower	Upper
0.0	-0.29980	-0.29904
0.5	-0.10118	-0.10062
1.0	-0.04923	-0.04876
1.5	-0.02354	-0.02312
2.0	-0.00888	-0.00849
2.5	-0.00111	-0.00076
3.0	0.00326	0.00358
3.5	0.00583	0.00614
4.0	0.00727	0.00755
4.5	0.00804	0.00830
5.0	0.00832	0.00857
5.5	0.00849	0.00871
6.0	0.00851	0.00872
6.5	0.00815	0.00835
7.0	0.00819	0.00838
7.5	0.00795	0.00814
8.0	0.00749	0.00766
8.5	0.00718	0.00734
9.0	0.00675	0.00691
9.5	0.00640	0.00655
10.0	0.00605	0.00620

Table 3. Monte Carlo confidence intervals for the difference between multiple imputation estimates and true causal effects across efavirenz concentrations under G_{alt1}

COMPETING INTERESTS

No competing interest is declared.

AUTHOR CONTRIBUTIONS STATEMENT

The study was designed by MS, PD and HM. Data was collected and interpreted by PD, HM. All authors reviewed the study design and interpreted the data. Methods development was lead by AH. The first draft of the article was written by AH. All authors reviewed this and the following drafts, revising it critically for its content. All authors have read and approved the final version and agree with the manuscript's conclusion.

ACKNOWLEDGMENTS

We are grateful for the support of the CHAPAS-3 trial team, their advice regarding the data analysis and making their data available to us. We would like to thank David Burger, Sarah Walker, Di Gibb and Andrzej Bienczak for their help in interpreting the data. We thank Elizabeth Kaudha and Victor Musiime for discussing reasons for missingness with us. We would further like to acknowledge the help of Alexander Szubert in constructing some of the variables. Michael Schomaker is supported by the German Research Foundations (DFG) Heisenberg Programm (grants 465412241 and 465412441). The CHAPAS-3 trial was funded by the European Developing Countries Clinical Trials Partnership (IP.2007.33011.006), Medical Research Council UK (MC_UU_00004/03), Department for International Development UK, and Ministerio de Sanidady Consumo Spain. Cipla Ltd donated first-line antiretrovirals. We thank all the children, carers, and staff from all the centres participating in the CHAPAS-3 trial.

REFERENCES

A. Bienczak, P. Denti, A. Cook, L. Wiesner, V. Mulenga, C. Kityo, A. Kekitiinwa, D. M. Gibb, D. Burger, A. S. Walker, and H. McIlleron. Plasma efavirenz exposure, sex, and age predict virological response in hiv-infected african children. *Journal of Acquired Immune Deficiency Syndromes*, 73(2):161–168, 2016. URL `<GotoISI>://MEDLINE:27116047`.

M. Hernán and J. Robins. *Causal Inference: What If*. Chapman & Hall/CRC, Boca Raton, 2020.

M. Hernan and J. Robins. *Causal inference*. Chapman & Hall/CRC, Boca Raton, 2020. URL `https://www.hsph.harvard.edu/miguel-hernan/causal-inference-book/`.

M. Hernán and J. Robins. Estimating causal effects from epidemiologic data. *Journal of epidemiology and community health*, 60:578–86, 08 2006. doi: 10.1136/jech.2004.029496.

J. Honaker, G. King, and M. Blackwell. Amelia ii: A program for missing data. *Journal of Statistical Software*, 45(7):1–47, 2011.

K. Mohan and J. Pearl. Graphical models for recovering probabilistic and causal queries from missing data. In Z. Ghahramani, M. Welling, C. Cortes, N. Lawrence, and K. Weinberger, editors, *Advances in Neural Information Processing Systems*, volume 27. Curran Associates, Inc., 2014. URL `https://proceedings.neurips.cc/paper/2014/file/31839b036f63806cba3f47b93af8ccb5-Paper.pdf`.

K. Mohan and J. Pearl. Graphical models for processing missing data. *Journal of the American Statistical Association*, 116(534):1023–1037, 2021. doi: 10.1080/01621459.2021.1874961. URL `https://doi.org/10.1080/01621459.2021.1874961`.

K. Mohan, J. Pearl, and J. Tian. Graphical models for inference with missing data. In C. Burges, L. Bottou, M. Welling, Z. Ghahramani, and K. Weinberger, editors, *Advances in Neural Information Processing Systems*, volume 26. Curran Associates, Inc., 2013. URL `https://proceedings.neurips.cc/paper/2013/file/0ff8033cf9437c213ee13937b1c4c455-Paper.pdf`.

M. Moreno-Betancur, K. J. Lee, F. P. Leacy, I. R. White, J. A. Simpson, and J. B. Carlin. Canonical Causal Diagrams to Guide the Treatment of Missing Data in Epidemiologic Studies. *American Journal of Epidemiology*, 187(12):2705–2715, 08 2018. ISSN 0002-9262. doi: 10.1093/aje/kwy173. URL `https://doi.org/10.1093/aje/kwy173`.

V. Mulenga, V. Musiime, A. Kekitiinwa, A. D. Cook, G. Abongomera, J. Kenny, C. Chabala, G. Mirembe, A. Asiimwe, E. Owen-Powell, D. Burger, H. McIlleron, N. Klein, C. Chintu, M. J. Thomason, C. Kityo, A. S. Walker, and D. Gibb. Abacavir, zidovudine, or stavudine as paediatric tablets for african hiv-infected children (chapas-3): an open-label, parallel-group, randomised controlled trial. *The Lancet Infectious Diseases*, 16 (2):169–79, 2016.

J. Pearl. *Causality*. Cambridge University Press, 2 edition, 2009. doi: 10.1017/CBO9780511803161.

J. Robins. A new approach to causal inference in mortality studies with a sustained exposure period—application to control of the healthy worker survivor effect. *Mathematical Modelling*, 7(9): 1393–1512, 1986. ISSN 0270-0255. doi: https://doi.org/10.1016/0270-0255(86)90088-6. URL `https://www.sciencedirect.com/science/article/pii/0270025586900886`.

D. B. Rubin. Inference and missing data. *Biometrika*, 63(3):581–592, 1976. ISSN 00063444. URL `http://www.jstor.org/stable/2335739`.

J. L. Schafer and J. W. Graham. Missing data: our view of the state of the art. *Psychological methods*, 7 2: 147–77, 2002.

M. Schomaker. Regression and causality, 2020. URL `https://arxiv.org/abs/2006.11754`.

M. Schomaker, S. Hogger, L. F. Johnson, C. J. Hoffmann, T. Barnighausen, and C. Heumann. Simultaneous treatment of missing data and measurement error in hiv research using multiple overimputation. *Epidemiology*, 26(5):628–636, 2015.

M. Schomaker, H. McIlleron, P. Denti, and I. Dìaz. Causal inference with continuous multiple time point interventions. *arXiv e-prints*, https://arxiv.org/abs/2305.06645, 2023.

S. Seaman, J. Galati, D. Jackson, and J. Carlin. What is meant by "missing at random"? *Statistical Science*, 28(2), may 2013. doi: 10.1214/13-sts415. URL `https://doi.org/10.1214%2F13-sts415`.

I. Shpitser and J. Pearl. Identification of joint interventional distributions in recursive semi-markovian causal models. In *Proceedings of the 21st National Conference on Artificial Intelligence - Volume 2*, AAAI'06, page 1219–1226. AAAI Press, 2006. ISBN 9781577352815.

O. Sofrygin, M. J. van der Laan, and R. Neugebauer. simcausal R package: Conducting transparent and reproducible simulation studies of causal effect estimation with complex longitudinal data. *Journal of Statistical Software*, 81(2):1–47, 2017. doi: 10.18637/jss.v081.i02.

J. Tian. Missing at Random in Graphical Models. In G. Lebanon and S. V. N. Vishwanathan, editors, *Proceedings of the Eighteenth International Conference on Artificial Intelligence and Statistics*, volume 38 of *Proceedings of Machine Learning Research*, pages 977–985, San Diego, California, USA, 09–12 May 2015. PMLR. URL `https://proceedings.mlr.press/v38/tian15.html`.

J. Tian and J. Pearl. A general identification condition for causal effects. In *Eighteenth National Conference on Artificial Intelligence*, page 567–573, USA, 2002. American Association for Artificial Intelligence. ISBN 0262511290.

S. Tikka and J. Karvanen. Identifying causal effects with the R package causaleffect. *Journal of Statistical Software*, 76(12):1–30, 2017. doi: 10.18637/jss.v076.i12.

M. van der Laan and S. Rose. *Targeted Learning: Causal Inference for Observational and Experimental Data*. Springer Series in Statistics. Springer New York, 2011. ISBN 9781441997821. URL `https://books.google.de/books?id=RGnSX5aCAgQC`.
