# Supplementary figures and images for: Recoverability of causal effects under presence of missing data: a longitudinal case study
Source: Biostatistics. 2024 Nov 16;26(1):kxae044. doi: 10.1093/biostatistics/kxae044 (PMC7617375; doi:10.1093/biostatistics/kxae044)

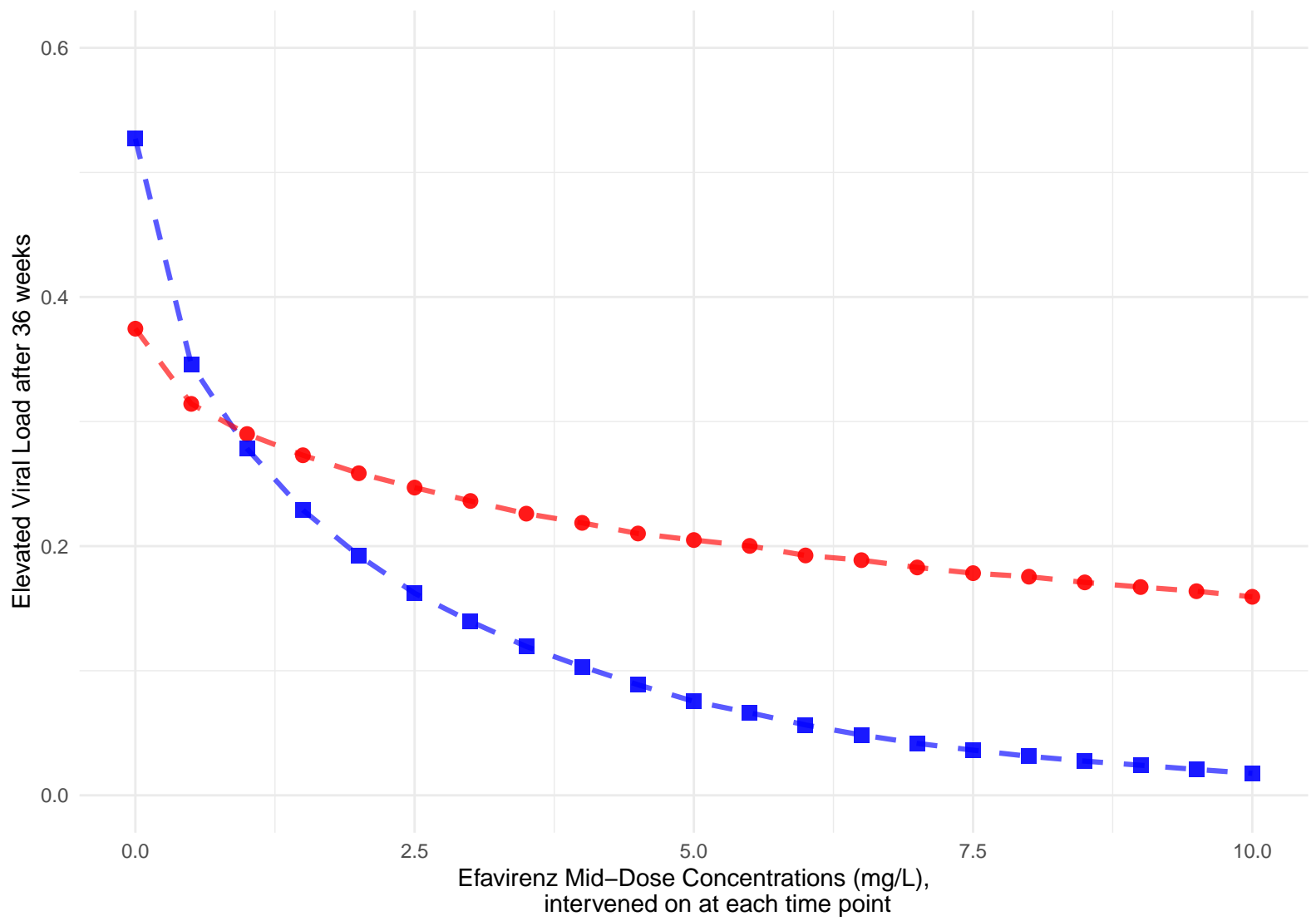

Supplement: kxae044_Supplementary_Data [file kxae044_supplementary_data.zip › 1650117mhjtpfkghzcddgmsdgnjbrrdmftrfsbq/plots/chapas3_vl36.pdf]

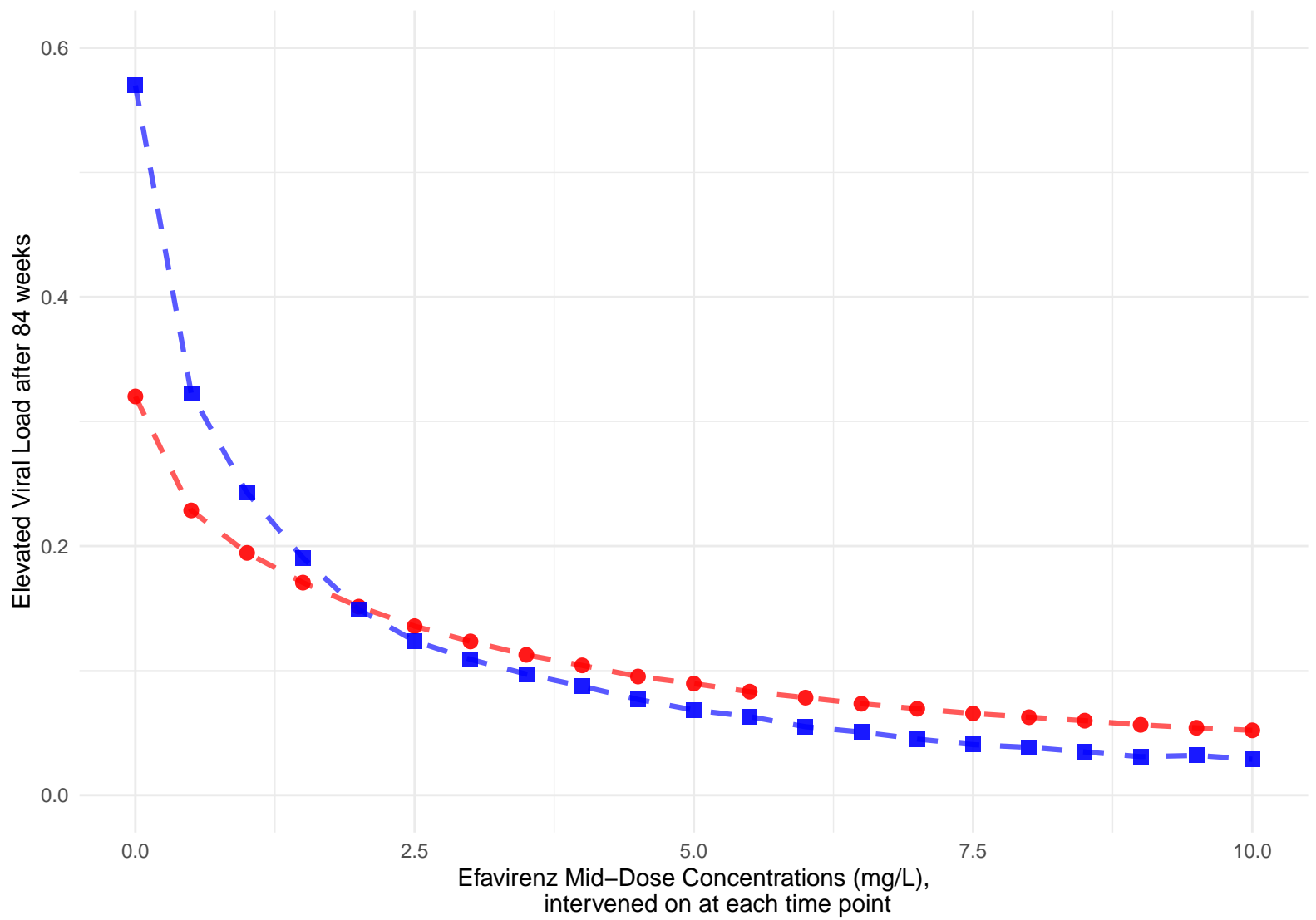

Supplement: kxae044_Supplementary_Data [file kxae044_supplementary_data.zip › 1650117mhjtpfkghzcddgmsdgnjbrrdmftrfsbq/plots/chapas3_vl84.pdf]

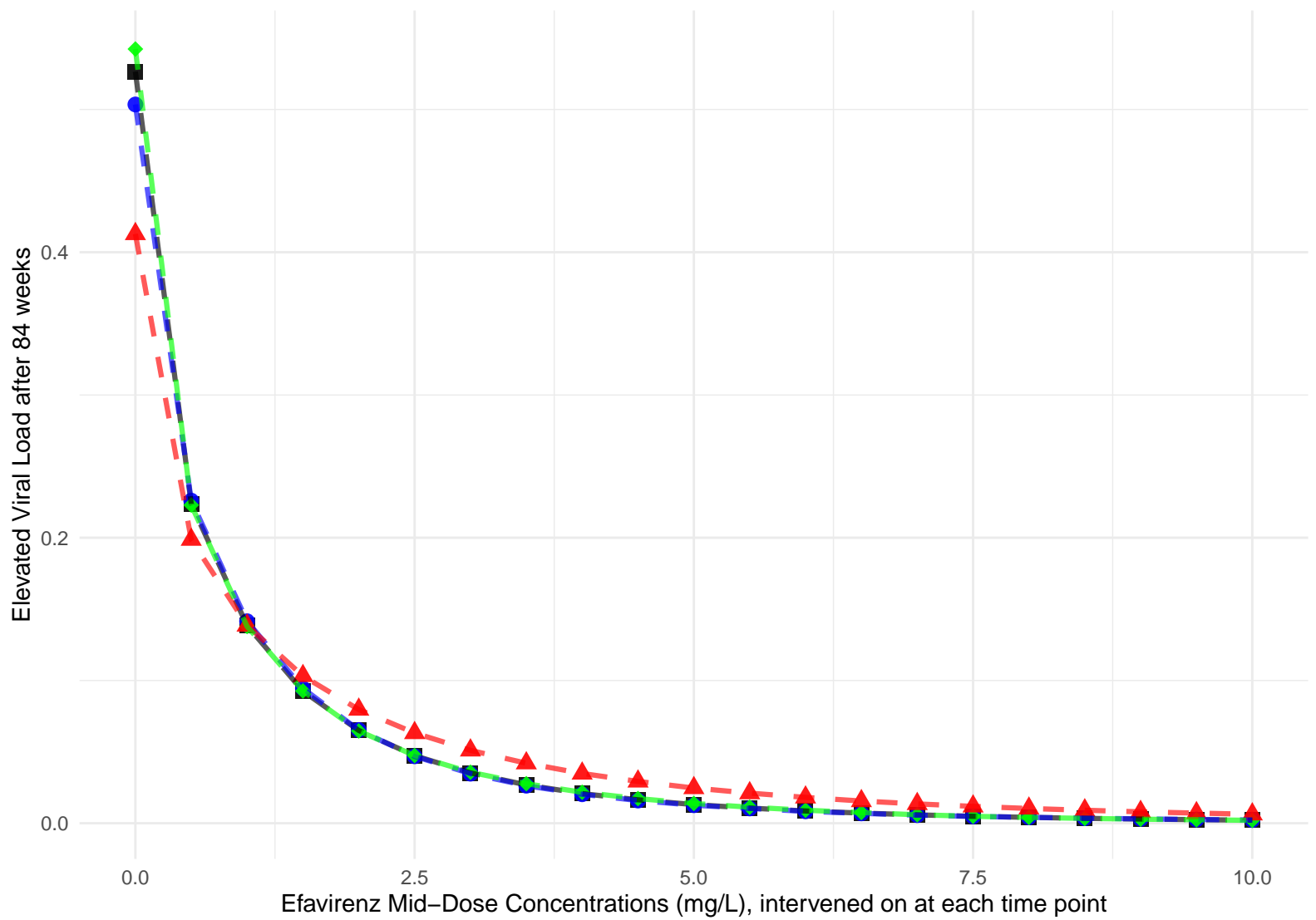

Supplement: kxae044_Supplementary_Data [file kxae044_supplementary_data.zip › 1650117mhjtpfkghzcddgmsdgnjbrrdmftrfsbq/plots/vl84_altern2_2miss.pdf]

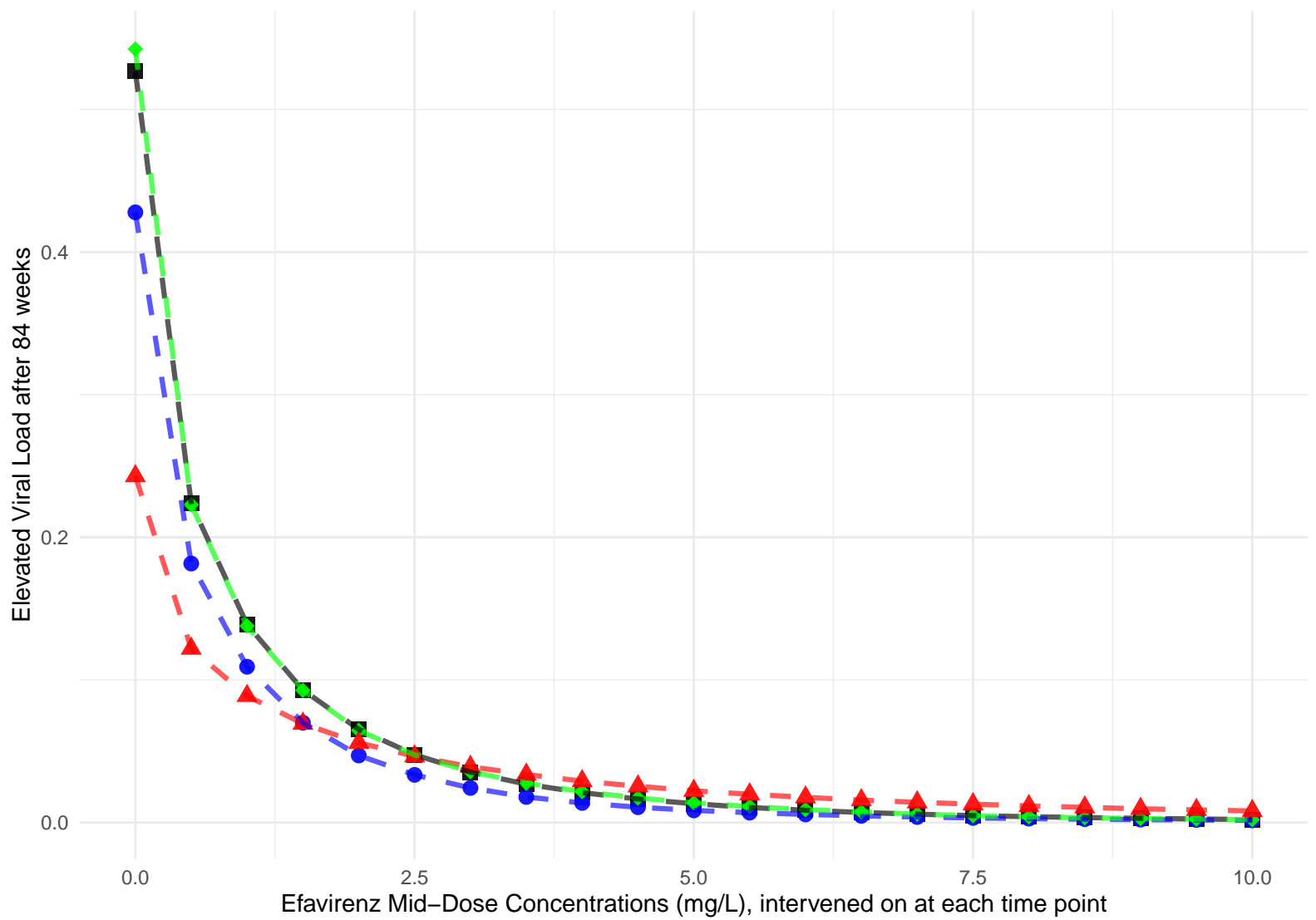

Supplement: kxae044_Supplementary_Data [file kxae044_supplementary_data.zip › 1650117mhjtpfkghzcddgmsdgnjbrrdmftrfsbq/plots/vl84_altern_2miss.pdf]

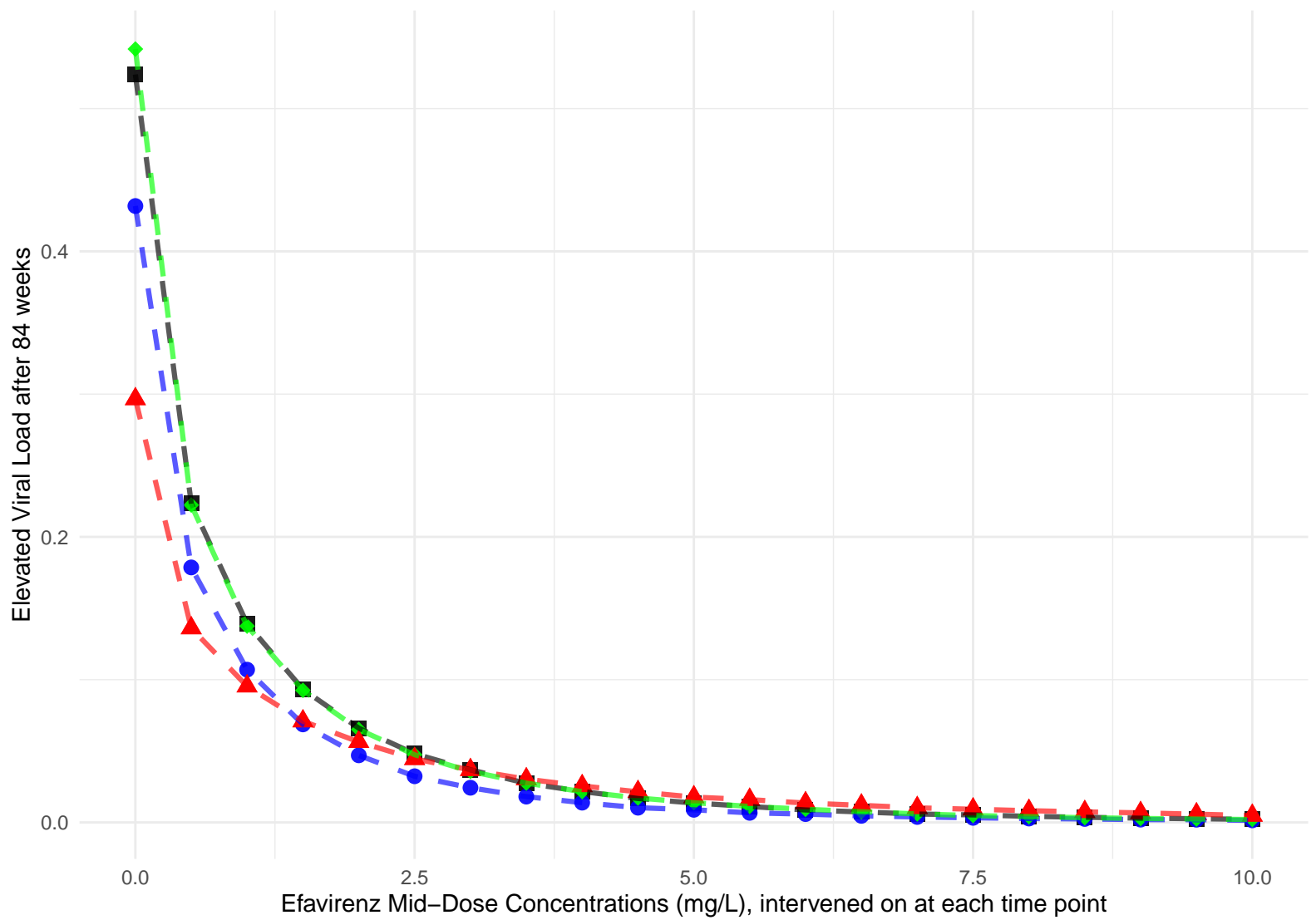

Supplement: kxae044_Supplementary_Data [file kxae044_supplementary_data.zip › 1650117mhjtpfkghzcddgmsdgnjbrrdmftrfsbq/plots/vl84_altern_4miss.pdf]

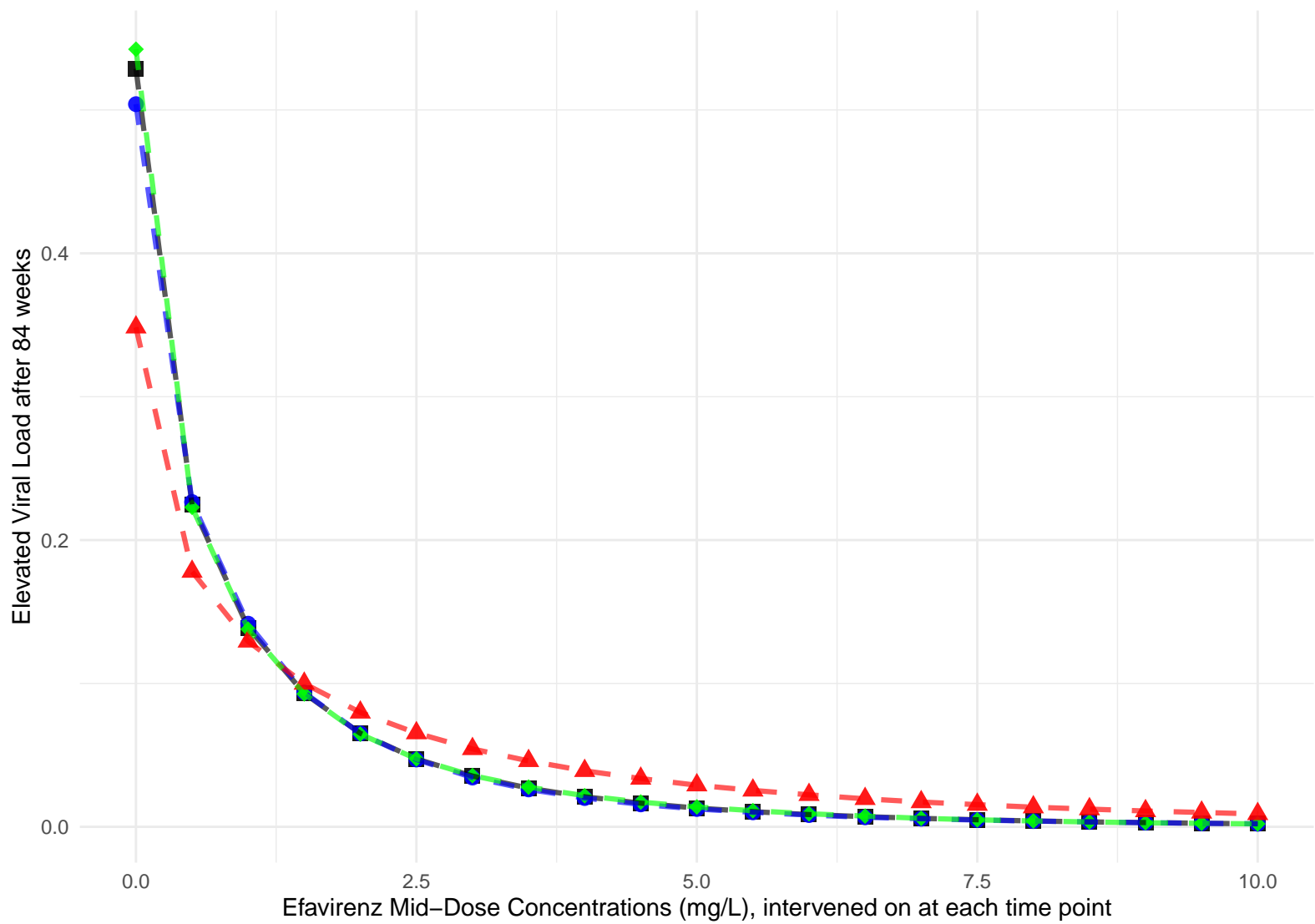

Supplement: kxae044_Supplementary_Data [file kxae044_supplementary_data.zip › 1650117mhjtpfkghzcddgmsdgnjbrrdmftrfsbq/plots/vl84_main_2miss.pdf]

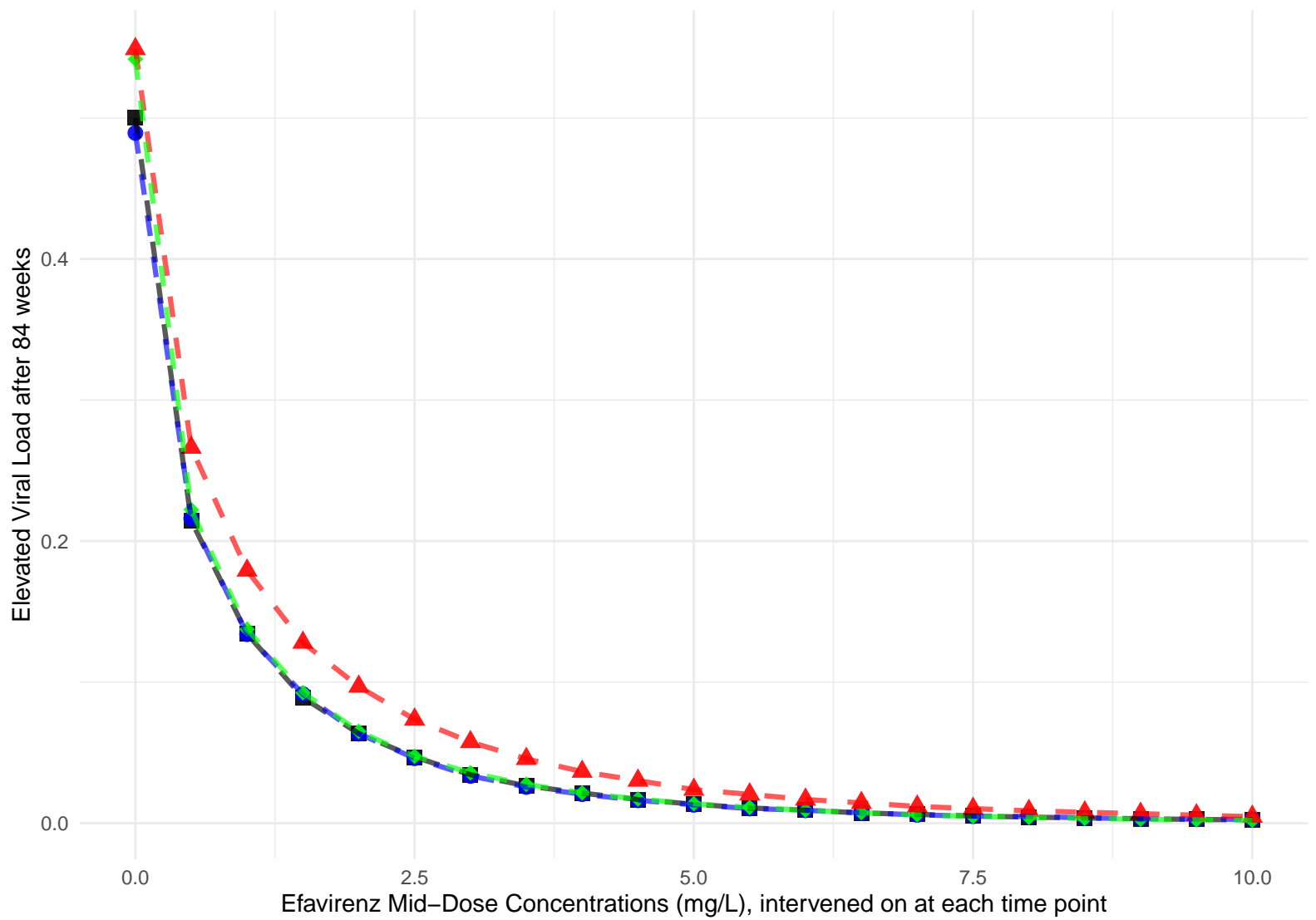

Supplement: kxae044_Supplementary_Data [file kxae044_supplementary_data.zip › 1650117mhjtpfkghzcddgmsdgnjbrrdmftrfsbq/plots/vl84_main_4miss.pdf]
