# Supplementary material for: Recoverability of causal effects under presence of missing data: a longitudinal case study
Source: Biostatistics. Author manuscript; Available in PMC 2025 Feb 5. (PMC7617375; doi:10.1093/biostatistics/kxae044)
Supplement: Supplementary file 1 [file EMS202828-supplement-Appendix.pdf]

Downloaded from https://academic.oup.com/biostatistics/advance-article/doi/10.1093/biostatistics/kxae044/7902044 by guest on 24 January 2025

A. STRUCTURAL EQUATION MODEL

We now present the non-parametric structural equation models (SEMs) corresponding to the m-DAGs in Fig. 3. Note that the follow-up time points 1 through 5 coincide with the study weeks 6, 36, 48, 60, and 84. The independent (joint) noise term is denoted as **U**.

The SEM corresponding to $\mathbf{G}_{main}$ in Fig. 3 is as follows:
For $t = 0$:

$$\begin{aligned}
\text{Genotype} &= f_{\text{Genotype}}(\text{Sex}, U_{\text{Genotype}}) \\
\text{Weight}_0 &= f_{\text{Weight}_0}(\text{Sex}, \text{Age}, U_{\text{Weight}_0}) \\
\text{NRTI}_0 &= f_{\text{NRTI}}(\text{Age}, U_{\text{NRTI}}) \\
\text{MV}_0 &= f_{\text{MV}_0}(\text{BMQ}, \text{SES}, \text{BHV}, U_{\text{MV}_0})
\end{aligned}$$

For $t = 1$:

$$\text{Dose}_1 = f_{\text{Dose}_1}(\text{Weight}_1, U_{\text{Dose}_1})$$

For $t \geq 0$:

$$\begin{aligned}
\text{M}_{Weight_t} &= f_{\text{M}_{Weight_t}}(\text{MV}_t, U_{\text{M}_{Weight_t}}) \\
\text{M}_{VL_t} &= f_{\text{M}_{VL_t}}(\text{MV}_t, \text{TI}_t, U_{\text{M}_{VL_t}})
\end{aligned}$$

For $t \geq 1$:

$$\begin{aligned}
\text{MEMS}_t &= f_{\text{MEMS}_t}(\text{CoMo}_{t-1}, \text{MEMS}_{t-1}, U_{\text{MEMS}_t}) \quad [\text{assume MEMS}_0 = 0] \\
\text{Weight}_t &= f_{\text{Weight}_t}(\text{Weight}_{t-1}, \text{CoMo}_{t-1}, U_{\text{Weight}_t}) \\
\text{CoMo}_t &= f_{\text{CoMo}_t}(\text{CoMo}_{t-1}, \text{Age}, \text{Weight}_{t-1}, \text{VL}_{t-1}, U_{\text{CoMo}_t}) \\
\text{EFV}_t &= f_{\text{EFV}_t}(\text{Dose}_t, \text{MEMS}_t, \text{Genotype}, U_{\text{EFV}_t}) \\
\text{VL}_t &= f_{\text{VL}_t}(\text{VL}_{t-1}, \text{CoMo}_{t-1}, \text{EFV}_t, U_{\text{VL}_t}) \\
\text{MV}_t &= f_{\text{MV}_t}(\text{BMQ}, \text{SES}, \text{BHV}, U_{\text{MV}_t}) \\
\text{M}_{EFV_t} &= f_{\text{M}_{EFV_t}}(\text{MV}_t, \text{TI}_t, U_{\text{M}_{EFV_t}}) \\
\text{M}_{MEMS_t} &= f_{\text{M}_{MEMS_t}}(\text{TI}_t, U_{\text{M}_{MEMS_t}})
\end{aligned}$$

For $t \geq 2$:

$$\text{Dose}_t \;= f_{\text{Dose}_t}(\text{Dose}_{t-1}, \text{Weight}_t, U_{\text{Dose}_t})$$

The SEM for $\mathbf{G}_{alt1}$ (with present blue dashed lines in Fig. 3) is the same as the SEM above, except for the structural equations for MV_t, $t \in \{0, 6, 36, 48, 60, 84\}$. These are specified as follows for $\mathbf{G}_{alt1}$:

For $t = 0$:

$$\text{MV}_0 \;= f_{\text{MV}_0}(\text{BMQ}, \text{SES}, \text{BHV}, \text{VL}_0, U_{\text{MV}_0})$$

For $t \geq 1$:

$$\text{MV}_t \;= f_{\text{MV}_t}(\text{BMQ}, \text{SES}, \text{BHV}, \text{VL}_t, U_{\text{MV}_t})$$

This way, MV_t additionally depends on VL_t, $t \in \{0, 6, 36, 48, 60, 84\}$, which corresponds to the dashed blue lines in the DAG.

B. IDENTIFIABILITY RESULTS

B.1. Identifiability results for θ_{84}

The identifiability result below is based on application of the IDC algorithm (Shpitser and Pearl 2006).

$$\begin{aligned}
\theta_{84} = \sum_{\substack{Age,\, Sex,\, CoMo_0,\, Weight_0,\\ MEMS_6, Weight_6, VL_0, CoMo_6,\\ VL_6,\, MEMS_{36},\, Weight_{36},\, CoMo_{36},\\ MEMS_{48}, Weight_{48}, VL_{36}, CoMo_{48},\\ VL_{48}, CoMo_{60}, VL_{60}}} & P(VL_{84} = 1 | Age, Sex, CoMo_0, Weight_0, Genotype, MEMS_6, Weight_6,\\
& Dose_6, EFV_6 = a, VL_0, CoMo_6, VL_6, MEMS_{36},\\
& Weight_{36}, CoMo_{36}, Dose_{36}, MEMS_{48}, Weight_{48}, EFV_{36} = a,\\
& Dose_{48}, VL_{36}, EFV_{48} = a, CoMo_{48}, VL_{48}, MEMS_{60}, Weight_{60}, CoMo_{60},\\
& Dose_{60}, MEMS_{84}, Weight_{84}, EFV_{60} = a, Dose_{84}, VL_{60}, EFV_{84} = a)\\
& P(VL_{60} | Age, Sex, CoMo_0, Weight_0, Genotype, MEMS_6, Weight_6,\\
& Dose_6, EFV_6 = a, VL_0, CoMo_6, VL_6, MEMS_{36},\\
& Weight_{36}, CoMo_{36}, Dose_{36}, MEMS_{48}, Weight_{48}, EFV_{36} = a, Dose_{48},\\
& VL_{36}, EFV_{48} = a, CoMo_{48}, VL_{48}, MEMS_{60}, Weight_{60}, Dose_{60}, EFV_{60} = a)\\
& P(CoMo_{60} | Age, Sex, CoMo_0, Weight_0, Genotype, MEMS_6, Weight_6,\\
& Dose_{36}, Dose_6, EFV_6 = a, VL_0, CoMo_6, VL_6, MEMS_{36}, Weight_{36}, CoMo_{36},\\
& MEMS_{48}, Weight_{48}, EFV_{36} = a, Dose_{48}, VL_{36}, EFV_{48} = a, CoMo_{48}, VL_{48})\\
& P(VL_{48} | Age, Sex, CoMo_0, Weight_0, Genotype, MEMS_6, Weight_6, Dose_6,\\
& EFV_6 = a, VL_0, CoMo_6, VL_6, MEMS_{36}, Weight_{36}, CoMo_{36}, Dose_{36},\\
& MEMS_{48}, Weight_{48}, EFV_{36} = a, Dose_{48}, VL_{36}, EFV_{48} = a)\\
& P(CoMo_{48} | Age, Sex, CoMo_0, Weight_0, Genotype, MEMS_6, Weight_6,\\
& Dose_6, EFV_6 = a, VL_0, CoMo_6, VL_6, MEMS_{36},\\
& Weight_{36}, CoMo_{36}, Dose_{36}, EFV_{36} = a, VL_{36})
\end{aligned}$$

Downloaded from https://academic.oup.com/biostatistics/advance-article/doi/10.1093/biostatistics/kxae044/7902044 by guest on 24 January 2025

$$P(Weight_{48}|Age, Sex, CoMo_0, Weight_0, Genotype, MEMS_6, Weight_6,$$
$$Dose_6, EFV_6 = a, VL_0, CoMo_6, VL_6, Weight_{36}, CoMo_{36})$$
$$P(MEMS_{48}|Age, Sex, CoMo_0, Weight_0, Genotype, MEMS_6, Weight_6,$$
$$Dose_6, EFV_6 = a, VL_0, CoMo_6, VL_6, MEMS_{36}, CoMo_{36})$$
$$P(CoMo_{36}|Age, Sex, CoMo_0, Weight_0, Genotype, MEMS_6, Weight_6,$$
$$Dose_6, EFV_6 = a, VL_0, CoMo_6, VL_6)$$
$$P(VL_{36}|Age, Sex, CoMo_0, Weight_0, Genotype, MEMS_6, Weight_6, Dose_6,$$
$$EFV_6 = a, VL_0, CoMo_6, VL_6, MEMS_{36}, Weight_{36}, Dose_{36}, EFV_{36} = a)$$
$$P(Weight_{36}|Age, Sex, CoMo_0, Weight_0, Genotype, Weight_6,$$
$$VL_0, CoMo_6)P(MEMS_{36}|Age, Sex, CoMo_0, Weight_0, Genotype, MEMS_6,$$
$$VL_0, CoMo_6)P(VL_6|Age, Sex, CoMo_0, Weight_0, Genotype,$$
$$MEMS_6, Weight_6, Dose_6, EFV_6 = a, VL_0)$$
$$P(CoMo_6|Age, Sex, CoMo_0, Weight_0, Genotype, VL_0)$$
$$P(Weight_6|Age, Sex, CoMo_0, Weight_0)P(MEMS_6|CoMo_0)$$
$$P(Weight_0|Age, Sex)P(CoMo_0|Age, Sex)P(VL_0)P(Sex)P(Age) \quad (5)$$

An alternative identifiability result can be obtained, for example, by applying the generalized back-door criterion. This results in using an adjustment set given by the confounders weight and adherence (MEMS). Both dose and co-morbidities are not necessarily required to block the relevant back-door paths from EFV_t to $VL_{t^*}, t^* \geq t$, i.e. the back-door paths that do not pass through any future concentrations (Hernán and Robins 2020a). Applying Robins' parametric g-formula (Robins 1986; Hernán and Robins 2020a) leads to the following valid g-formula factorization:

$$\theta_{84} = \int_l \Bigg\{ P(VL_{84} = 1|\bar{A}_{84} = \bar{a}_{84}, \bar{\mathbf{L}}_{84} = \bar{\mathbf{l}}_{84}) \times \prod_{s=0}^{84} f(\mathbf{L}_s = \mathbf{l}_s|\bar{A}_{s-1} = \bar{a}_{s-1}, \bar{\mathbf{L}}_{s-1} = \bar{\mathbf{l}}_{s-1}) \Bigg\} \, dF_l(l)\,, \quad (6)$$

where $F_L(\cdot)$ denotes the CDF with respect to L. In the above, $\mathbf{L}_t = \{\text{weight}_t, \text{MEMS}_t\}$, $\mathbf{L}_0 = \{\text{weight}_0, \text{NRTI, Genotype, Sex, Age}\}$ and $A_t = \text{EFV}_t$. Note that the overbar refers to the intervention and confounder histories, i.e. for of a unit i (up to and including time t) the histories are $\bar{A}_{t,i} = (A_{0,i}, \ldots, A_{t,i})$ and $\bar{L}^s_{t,i} = (L^s_{0,i}, \ldots, L^s_{t,i})$, $s = 1, 2$, $i = 1, \ldots, n$, respectively. Outcomes (i.e. VL) prior to s are part of $\bar{\mathbf{L}}_s$. The inner product of the 2 time-varying and ordered confounders $\mathbf{L}_s = \{L^1_s, L^2_s\}$ can be decomposed further:

$$\prod_{s=0}^{84} f(L^2_s = l^2_s \mid L^1_s = l^1_s, \bar{A}_{s-1} = \bar{a}_{s-1}, \bar{L}_{s-1} = \bar{\mathbf{l}}_{s-1}) \times f(L^1_s = l^1_s \mid \bar{A}_{s-1} = \bar{a}_{s-1}, \bar{\mathbf{L}}_{s-1} = \bar{\mathbf{l}}_{s-1})$$

Thus, implementing (6), requires fitting models for the outcome and two confounder distributions at each time point, given their respective history.

Downloaded from https://academic.oup.com/biostatistics/advance-article/doi/10.1093/biostatistics/kxae044/7902044 by guest on 24 January 2025

C. RECOVERABILITY RESULTS FOR θ_{36}

C.1. Assuming $\mathbf{G_{main}}$

To assess the recoverability of the identified causal effect θ_{36} in Equation 3, we must show that each of the multiplicative factors corresponding to the conditional or marginal distributions can be expressed in terms of the observed data only. Although the missingness mechanism is of the "closed" type, and the recoverability result is directly available for this case, we want to demonstrate how one would examine identifiability if such a result is not available. T do so, we present the result for one of the conditional distributions containing partially observed variables. The results for other multiplicative factors are derived analogously.

$$\begin{aligned}
&P(CoMo_6^{M=0}|Age, Sex, CoMo_0^{M=0}, Weight_0^{M=0}, Genotype, VL_0^{M=0})\\
&\quad= P(CoMo_6^{M=0}|Age, Sex, CoMo_0^{M=0}, Weight_0^{M=0}, Genotype, VL_0^{M=0}, \mathbf{M} = 0)\\
&\quad= P(CoMo_6|Age, Sex, CoMo_0, Weight_0, Genotype, VL_0, \mathbf{M} = 0)
\end{aligned}$$

The first equality holds due to the fact that all partially observed variables are independent of the corresponding relevant missingness indicators, whereas the second equality is true due to the consistency assumption (Moreno-Betancur et al. 2018).

Note that the recoverability result above is trivial due to the specific missingness mechanism. In a general case, much more effort is required to recover a distribution of interest.

C.2. Assuming $\mathbf{G_{alt2}}$

We consider a second plausible alternative m-DAG, $\mathbf{G}_{alt2}$, which is equivalent to $\mathbf{G}_{main}$ in Fig. 3, with the addition of a direct effect of *BHV* on *MEMS* for weeks 6, 36, 48, 60 and 84. In this model, we propose that behavioral pattern may affect adherence.

Under $\mathbf{G}_{alt2}$, the identified expression for the causal effect of interest, θ_{36}, is as follows:

$$\begin{aligned}
\theta_{36} = \sum_{\substack{Age,\,Sex,\,CoMo_0,\,Weight_0,\\ VL_0,\,CoMo_6,\,VL_6}} &P(VL_{36}|Age, Sex, BHV, CoMo_0, Weight_0, Genotype, MEMS_6,\\
&Weight_6, Dose_6, EFV_6 = a, VL_0, CoMo_6, VL_6, MEMS_{36}, Weight_{36},\\
&Dose_{36}, EFV_{36} = a)\\
&P(VL_6|Age, Sex, CoMo_0, Weight_0, Genotype, MEMS_6,\\
&Weight_6, Dose_6, EFV_6 = a, VL_0)\\
&P(CoMo_6|Age, Sex, CoMo_0, Weight_0, Genotype, VL_0)\\
&P(Weight_0|Age, Sex)P(CoMo_0|Age, Sex)P(VL_0)P(Sex)P(Age).
\end{aligned} \tag{7}$$

Note that even if the identified expression looks almost identical to the one we obtain under $\mathbf{G}_{main}$ or $\mathbf{G}_{alt1}$ (compare Equation 3), the recoverability result may differ due to the different conditional independence statements that hold in $\mathbf{G}_{alt2}$, assuming it is faithful.

D. SIMULATION STUDIES

D.1. DGP for simulations 1 and 2

Both baseline data ($t = 0$) and follow-up data ($t = 1, \ldots, 5$) were created using structural equations with the *R*-package `simcausal`. Note that the follow-up time points 1 through 5 correspond to the study weeks 6, 36, 48, 60 and 84. The distributions listed below, in temporal order, describe the data-generating process. Our baseline data consists of *Sex*, *Genotype*, log(age) (*Age*), log(weight) (*Weight*), the respective Nucleoside Reverse Transcriptase Inhibitor (*NRTI*), and a proxy for

Downloaded from https://academic.oup.com/biostatistics/advance-article/doi/10.1093/biostatistics/kxae044/7902044 by guest on 24 January 2025

socio-economic status (SES). The time-varying variables are co-morbidities (CoMo), efavirenz dose (Dose), efavirenz mid-dose concentration (EFV), elevated viral load (= viral failure, VL), adherence (measured through memory caps, MEMS), missed visit (MV), technical issues (TI), and the missingness indicators for *EFV*, *Weight*, *VL*, and *MEMS*, respectively. In addition to Bernoulli (B), Poisson ($Poisson$), Multinominal (MN) and Normal (N) distributions, we also use truncated normal distributions, denoted by $N_{[a,a_1,a_2,b,b_1,b_2]}$, where a and b are the truncation levels. Values smaller than a are replaced by a random draw from a $U(a_1, a_2)$ distribution and values greater than b are drawn from a $U(b_1, b_2)$ distribution, where U refers to a continuous uniform distribution. For the specified multinomial distributions, probabilities are normalized, if required, to ensure they add up to 1.

The DGP corresponding to $\mathbf{G}_{main}$ in Fig. 3 is as follows:
For $t = 0$:

$$
\begin{aligned}
\text{Sex}_0 &\sim \text{B}(p = 0.5)\\
\text{Genotype}_0 &\sim \text{MN}\begin{pmatrix} p1 = 1/(1+\exp(-(-0.103 + \text{I}(\text{Sex}_0 = 1)\times 0.223 + \text{I}(\text{Sex}_0 = 0)\times 0.173))),\\ p2 = 1/(1+\exp(-(-0.086 + \text{I}(\text{Sex}_0 = 1)\times 0.198 + \text{I}(\text{Sex}_0 = 0)\times 0.214))),\\ p3 = 1/(1+\exp(-(-0.309 + \text{I}(\text{Sex}_0 = 1)\times 0.082 + \text{I}(\text{Sex}_0 = 0)\times 0.170)))\end{pmatrix}\\
\text{Age}_0 &\sim N_{[0.693,0.693,1,2.8,2.7,2.8]}(\mu = 1.501, \sigma = 0.369)\\
\text{Weight}_0 &\sim N_{[2.26,2.26,2.67,3.37,3.02,3.37]}(\mu = (1.5 + 0.2\times \text{Sex}_0 + 0.774\times \text{Age}_0)\times 0.94), \sigma = 0.369)\\
\text{NRTI}_0 &\sim \text{MN}\begin{pmatrix} p1 = 1/(1+\exp(-(-0.006 + \text{I}(\text{Age}_0 > 1.4563)\times \text{Age}_0 \times 0.1735 + \text{I}(\text{Age}_0 \le 1.4563)\times \text{Age}_0\times 0.1570))),\\ p2 = 1/(1+\exp(-(-0.006 + \text{I}(\text{Age}_0 > 1.4563)\times \text{Age}_0 \times 0.1735 + \text{I}(\text{Age}_0 \le 1.4563)\times \text{Age}_0\times 0.1570))),\\ p3 = 1/(1+\exp(-(-0.006 + \text{I}(\text{Age}_0 > 1.4563)\times \text{Age}_0 \times 0.1570 + \text{I}(\text{Age}_0 \le .14563)\times \text{Age}_0\times 0.1818)))\end{pmatrix}\\
\text{CoMo}_0 &\sim \text{B}(p = 0.15)\\
\text{VL}_0 &\sim \text{B}(p = 1 - (1/(1+\exp(-(0.4 + 1.9\times\sqrt{\text{EFV}_0})))))\\
\text{SES}_0 &\sim Poisson(\lambda = 3)\\
\text{MV}_0 &\sim \text{B}(p = 1/(1+\exp(-(-2.95 + 0.1\times \text{SES}_0))))
\end{aligned}
$$

For $t = 1$:

$$
\text{Dose}_1 \sim \text{MN}\begin{pmatrix} p1 = 1/(1+\exp(-(5 + \sqrt{(\text{Weight}_1)}\times 8 - \text{Age}_0\times 10))),\\ p2 = 1/(1+\exp(-(4 + \sqrt{(\text{Weight}_1)}\times 8.768 - \text{Age}_0\times 9.06))),\\ p3 = 1/(1+\exp(-(3 + \sqrt{(\text{Weight}_1)}\times 6.562 - \text{Age}_0\times 8.325))),\\ p4 = 1 - (p1 + p2 + p3)\end{pmatrix}
$$

For $t \geq 0$:

$$
\begin{aligned}
\text{TI}_t &\sim \text{B}(p = 0.05))))\\
\text{M}_{EFV_t} &\sim \text{B}(p = I(\text{MV}_t = 1) + I(\text{MV}_t = 0)\times I(\text{TI}_t = 1)\times 0.5))))\\
\text{M}_{Weight_t} &\sim \text{B}(p = I(\text{MV}_t = 1))\\
\text{M}_{VL_t} &\sim \text{B}(p = I(\text{MV}_t = 1) + I(\text{MV}_t = 0)\times I(\text{TI}_t = 1)\times 0.5))))
\end{aligned}
$$

For $t \geq 1$:

$$
\begin{aligned}
\text{MEMS}_t &\sim \text{B}(p = 1/(1+\exp(-(0.71 + \text{CoMo}_{t-1}\times 0.31 + \text{MEMS}_{t-1}\times \text{I}(t\geq 2)\times 0.31)))), \quad [\text{assume MEMS}_0 = 0]\\
\text{Weight}_t &\sim N_{[2.26,2.26,2.473,3.37,3.2,3.37]}(\mu = \text{Weight}_{t-1}\times 1.04 - 0.05\times \text{I}(\text{CoMo}_{t-1} = 1), \sigma = 0.4)
\end{aligned}
$$

Downloaded from https://academic.oup.com/biostatistics/advance-article/doi/10.1093/biostatistics/kxae044/7902044 by guest on 24 January 2025

$$
\begin{aligned}
\text{CoMo}_t &\sim \text{B}(p = 1 - (1/(1 + \exp(-(0.5 \times \text{I}(\text{CoMo}_{t-1} = 1) + \text{Age}_0 \times 0.1 + \text{Weight}_{t-1} \times 0.1))))) \\
\text{EFV}_t &\sim N_{[0.2032,0.2032,0.88,21.84,8.37,21.84]}(\mu = 0.1 \times \text{Dose}_t + 0.1 \times \text{MEMS}_t + \text{I}(\text{Genotype}_0 \leq 2) \times 2.66 \\
&\quad + \text{I}(\text{Genotype}_0 = 3) \times 4.6, \sigma = 4.06) \\
\text{VL}_t &\sim \text{B}(p = 1 - (1/(1 + \exp(-(1 - 0.6 \times \text{I}(t = 1) - 1.2 \times \text{I}(t = 4) + 0.1 \times \text{CoMo}_{t-1} \\
&\quad + (2 - 0.2 \times \text{I}(t = 3)) \times \sqrt{\text{EFV}_t}))))) \\
\text{MV}_t &\sim \text{B}(p = 1/(1 + \exp(-(-2.95 + 0.1 \times \text{SES}_0 + \text{MV}_{t-1})))) \\
\text{M}_{MEMS_t} &\sim \text{B}(p = 1/(1 + \exp(-(0.5 \times I(\text{TI}_t = 1) + 0.2))))
\end{aligned}
$$

For $t \geq 2$:

$$
\text{Dose}_t \sim \text{MN}\begin{pmatrix} p1 = (1/(1 + \exp(-(4 + \text{Dose}_{t-1} \times 0.5 + \sqrt{\text{Weight}_t} \times 4 - \text{Age}_0 \times 10))), \\ p2 = (1/(1 + \exp(-(-8 + \text{Dose}_{t-1} \times 0.5 + \sqrt{\text{Weight}_t} \times 8.568 - \text{Age}_0 \times 9.06))), \\ p3 = (1/(1 + \exp(-(20 + \text{Dose}_{t-1} \times 0.5 + \sqrt{\text{Weight}_t} \times 6.562 - \text{Age}_0 \times 18.325))), \\ p4 = 1 - (p1 + p2 + p3) \end{pmatrix}
$$

The DGP for $\mathbf{G}_{alt1}$ (including the blue dashed lines in Fig. 3) coincides with the DGP above, except for the structural equations for MV_t, $t \in \{0, 6, 36, 48, 60, 84\}$. These are specified as follows for $\mathbf{G}_{alt1}$:

For $t = 0$:

$$
\text{MV}_0 \sim \text{B}(p = 1/(1 + \exp(-(-2.95 + 0.1 \times \text{SES}_0 + 2 \times \text{VL}_0))))
$$

For $t \geq 1$:

$$
\text{MV}_t \sim \text{B}(p = 1/(1 + \exp(-(-2.95 + 0.1 \times \text{SES}_0 + \text{MV}_{t-1} + 2 \times \text{VL}_t))))
$$

Thus, MV_t additionally depends on VL_t, $t \in \{0, 6, 36, 48, 60, 84\}$, which corresponds to the dashed blue lines in the DAG.

The DGP for $\mathbf{G}_{alt2}$ (where *SES* is a cause of $MEMS_t$, $t \in \{6, 36, 48, 60, 84\}$) coincides with the DGP for $\mathbf{G}_{main}$ above, except for the structural equations for $MEMS_t$, $t \in \{6, 36, 48, 60, 84\}$. These are specified for $\mathbf{G}_{alt2}$ as follows:
For $t \geq 1$:

$$
\begin{aligned}
\text{MEMS}_t \sim \text{B}(p = 1/(1 + \exp(-(0.71 + \text{CoMo}_{t-1} \times 0.31 + \text{MEMS}_{t-1} \\
\times \text{I}(t \geq 2) \times 0.31 - \text{SES}_0 \times 0.5))))
\end{aligned}
$$

After generating the data set using the structural equations, we introduce missing values based on the missingness indicators: if a missingness indicator equals 1, the corresponding covariate value is set to NA. In *Simulation 1*, we ignore the missingness indicators for EFV_t, $t \in \{6, 36, 48, 60, 84\}$, and $Weight_t$, $t \in \{0, 6, 36, 48, 60, 84\}$, and generate missingness only in VL_t and $MEMS_t$, $t \in \{0, 6, 36, 48, 60, 84\}$. In *Simulation 2*, missingness is introduced in EFV_t, $t \in \{6, 36, 48, 60, 84\}$, $Weight_t$, VL_t and $MEMS_t$, $t \in \{0, 6, 36, 48, 60, 84\}$.

D.2. Results

The results below are based on Simulation 2 as defined in Section 5.

The following results are based on simulation 1 as defined in Section 5 under the assumption of $\mathbf{G}_{alt2}$ being the true underlying causal m-DAG.

Downloaded from https://academic.oup.com/biostatistics/advance-article/doi/10.1093/biostatistics/kxae044/7902044 by guest on 24 January 2025

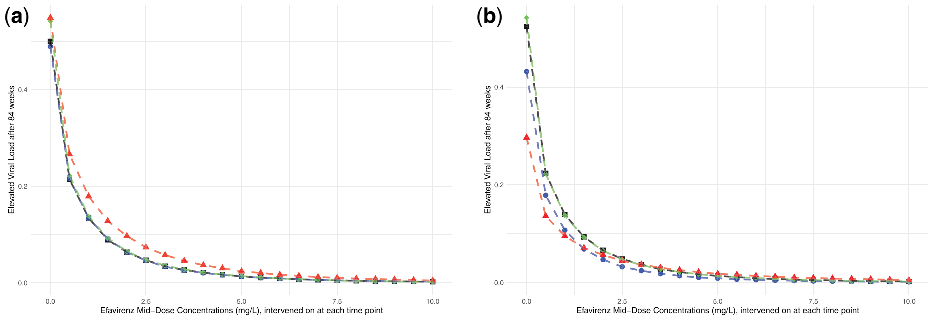

Data simulated under the main DAG $\mathbf{G}_{\text{main}}$ Data simulated under the alternative DAG $\mathbf{G}_{\text{alt1}}$

Figure D.1: Estimated CCRCs for the probability of viral failure after 84 wks. (a) Data simulated under the main DAG $\mathbf{G}_{\text{main}}$. (b) Data simulated under the alternative DAG $\mathbf{G}_{\text{alt1}}$. Causal effects were estimated on complete data (black squares), incomplete data using available cases (blue dots), incomplete data using multiple imputation (red triangles) and counterfactual data (green diamonds, true CCRC); results represent the mean over 1000 seeds.

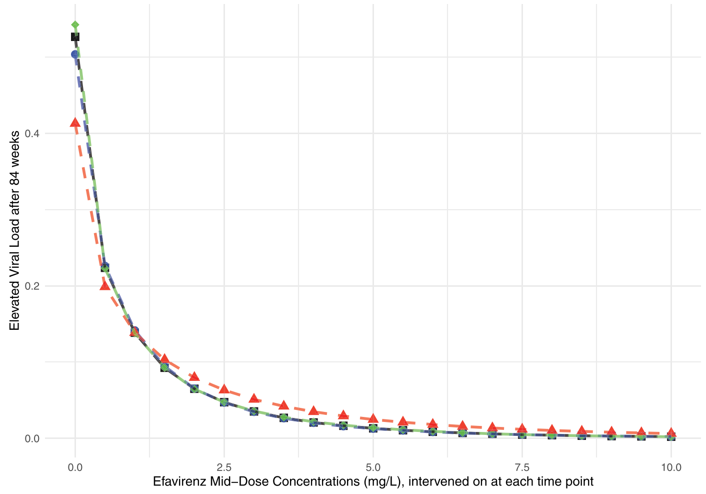

Figure D.2: Estimated CCRCs for the probability of viral failure after 84 wks based on data simulated under the main DAG $\mathbf{G}_{alt2}$. Causal effects were estimated on complete data (black squares), incomplete data using available cases (blue dots), incomplete data using multiple imputation (red triangles) and counterfactual data (green diamonds, true CCRC); results represent the mean over 1000 seeds.

D.3. Monte Carlo confidence intervals

The Monte Carlo confidence intervals in Tables D.1a, D.1b, and D.1c are reported to determine whether the differences between MI estimates and true values (main and alternative m-DAGs, $\mathbf{G}_{main}$ and $\mathbf{G}_{alt1}$), and between available case analysis estimates and true causal effects θ_{84} (alternative m-DAG $\mathbf{G}_{alt1}$) stem from simulation uncertainty or indicate systematic deviations. A 95% confidence interval [mean difference $-2 \cdot SE$; mean difference $+2 \cdot SE$], with the standard error SE computed as $sd(estimate)/\sqrt{(\#runs)}$, excluding zero suggests that the differences can likely not be explained by simulation uncertainty, indicating a bias caused by the estimation approach.

Downloaded from https://academic.oup.com/biostatistics/advance-article/doi/10.1093/biostatistics/kxae044/7902044 by guest on 24 January 2025

Table D.1. Monte Carlo confidence intervals for the difference between estimates and true causal effects across efavirenz (EFV) concentrations. (a) Multiple imputation estimates under the main m-DAG $\mathbf{G}_{\text{main}}$. (b) Available case estimates under the alternative m-DAG $\mathbf{G}_{\text{alt1}}$. (c) Multiple imputation (MI) estimates under the alternative m-DAG $\mathbf{G}_{\text{alt1}}$.

(a) Multiple imputation under $\mathbf{G}_{\text{main}}$

EFV	Lower	Upper
0.0	-0.19441	-0.19351
0.5	-0.04522	-0.04452
1.0	-0.00881	-0.00824
1.5	0.00696	0.00749
2.0	0.01493	0.01539
2.5	0.01774	0.01817
3.0	0.01842	0.01880
3.5	0.01812	0.01847
4.0	0.01721	0.01754
4.5	0.01626	0.01656
5.0	0.01502	0.01530
5.5	0.01403	0.01429
6.0	0.01309	0.01335
6.5	0.01191	0.01214
7.0	0.01123	0.01145
7.5	0.01051	0.01071
8.0	0.00952	0.00971
8.5	0.00886	0.00904
9.0	0.00810	0.00827
9.5	0.00750	0.00766
10.0	0.00685	0.00701

(b) Available case under $\mathbf{G}_{\text{alt1}}$

EFV	Lower	Upper
0.0	−0.12787	−0.10112
0.5	−0.04754	−0.03497
1.0	−0.03208	−0.02512
1.5	−0.02465	−0.02075
2.0	−0.01873	−0.01642
2.5	−0.01470	−0.01320
3.0	−0.01195	−0.01092
3.5	−0.01012	−0.00935
4.0	−0.00823	−0.00759
4.5	−0.00677	−0.00620
5.0	−0.00558	−0.00509
5.5	−0.00450	−0.00405
6.0	−0.00358	−0.00317
6.5	−0.00296	−0.00257
7.0	−0.00222	−0.00186
7.5	−0.00175	−0.00141
8.0	−0.00145	−0.00115
8.5	−0.00114	−0.00085
9.0	−0.00088	−0.00062
9.5	−0.00074	−0.00048
10.0	−0.00058	−0.00034

(c) Multiple imputation under $\mathbf{G}_{\text{alt1}}$

EFV	Lower	Upper
0.0	−0.29980	−0.29904
0.5	−0.10118	−0.10062
1.0	−0.04923	−0.04876
1.5	−0.02354	−0.02312
2.0	−0.00888	−0.00849
2.5	−0.00111	−0.00076
3.0	0.00326	0.00358
3.5	0.00583	0.00614
4.0	0.00727	0.00755
4.5	0.00804	0.00830
5.0	0.00832	0.00857
5.5	0.00849	0.00871
6.0	0.00851	0.00872
6.5	0.00815	0.00835
7.0	0.00819	0.00838
7.5	0.00795	0.00814
8.0	0.00749	0.00766
8.5	0.00718	0.00734
9.0	0.00675	0.00691
9.5	0.00640	0.00655
10.0	0.00605	0.00620

Downloaded from https://academic.oup.com/biostatistics/advance-article/doi/10.1093/biostatistics/kxae044/7902044 by guest on 24 January 2025
